# Intestinal stem cells contribute to the maturation of the neonatal small intestine and colon independently of digestive activity

**DOI:** 10.1038/s41598-017-09927-2

**Published:** 2017-08-31

**Authors:** Hirotsugu Yanai, Naho Atsumi, Toshihiro Tanaka, Naohiro Nakamura, Yoshihiro Komai, Taichi Omachi, Kiyomichi Tanaka, Kazuhiko Ishigaki, Kazuho Saiga, Haruyuki Ohsugi, Yoko Tokuyama, Yuki Imahashi, Shuichi Ohe, Hiroko Hisha, Naoko Yoshida, Keiki Kumano, Masanori Kon, Hiroo Ueno

**Affiliations:** 1 Department of Stem Cell Pathology, Kansai Medical University, 2-5-1 Shin-machi, Hirakata, Osaka, 573-1010 Japan; 2 Department of Surgery, Kansai Medical University, 2-5-1 Shin-machi, Hirakata, Osaka, 573-1010 Japan; 3 Third Department of Internal Medicine, Kansai Medical University, 2-5-1 Shin-machi, Hirakata, Osaka, 573-1010 Japan; 4 Department of Urology and Andrology, Kansai Medical University, 2-5-1 Shin-machi, Hirakata, Osaka, 573-1010 Japan; 5 Department of Pediatrics, Kansai Medical University, 2-5-1 Shin-machi, Hirakata, Osaka, 573-1010 Japan; 6 Department of Dermatology, Kansai Medical University, 2-5-1 Shin-machi, Hirakata, Osaka, 573-1010 Japan

## Abstract

The murine intestine, like that of other mammalians, continues to develop after birth until weaning; however, whether this occurs in response to an intrinsic developmental program or food intake remains unclear. Here, we report a novel system for the allotransplantation of small intestine and colon harvested from *Lgr5* ^*EGFP*-*IRES*-*CreERT2*/+^; *Rosa26* ^*rbw*/+^ mice immediately after birth into the subrenal capsule of wild-type mice. By histological and immunohistochemical analysis, the developmental process of transplanted small intestine and colon was shown to be comparable with that of the native tissues: mature intestines equipped with all cell types were formed, indicating that these organs do not require food intake for development. The intestinal stem cells in transplanted tissues were shown to self-renew and produce progeny, resulting in the descendants of the stem cells occupying the crypt-villus unit of the small intestine or the whole crypt of the colon. Collectively, these findings indicate that neonatal intestine development follows an intrinsic program even in the absence of food stimuli.

## Introduction

Intestinal tissues experience dynamic changes both in their structure and in the mechanisms of digestion and absorption, not only at birth but also at weaning^1^. External factors such as food intake may be involved in the maturation of intestines; however, internal factors are considered the dominant players in the maturation process. This notion has been deduced from the fact that the development of the intestines proceeds normally in infant rats that are maintained on a diet of milk instead of food after reaching the weaning age^1^. Several reports have supported this concept, establishing techniques for the transplantation of rat small intestines into the anterior chamber^2^ or subrenal capsule^3^, where the effects of digestion need not be considered. Nevertheless, it remains to be definitively confirmed whether the transplanted intestines proliferate in a stem cell-dependent manner as in the case of native tissues. Confirmation of such stem cell-dependent proliferation would demonstrate that food intake is not required for intestine maturation.

Here, we report on a murine allotransplantation system of intestinal tissues—both small intestine and colon—into the subrenal capsule. By transplanting neonatal intestines harvested from genetically engineered mice, which enables inducible multicolor lineage-tracing of Lgr5^+^ intestinal stem cells, the stem cell dynamics in the transplanted small intestine and colon were visualized. The development of the transplanted tissues was also assessed by histological and immunohistochemical analyses, which led to the identification of stem cells as a source of both self-renewing cells and other terminally differentiated cells—including Paneth cells and deep crypt secretory cells that function as the stem cell niche for the small intestine^4^ and colon^5^, respectively. These findings demonstrate the autonomous construction of self-contained intestinal tissues independently of food intake and the subsequent influences of digestion.

## Results

### Neonatal murine intestinal tissues exhibit adult-like turnover starting around postnatal day 7 and acquire all components comparable with mature tissues

**Figure 1:**
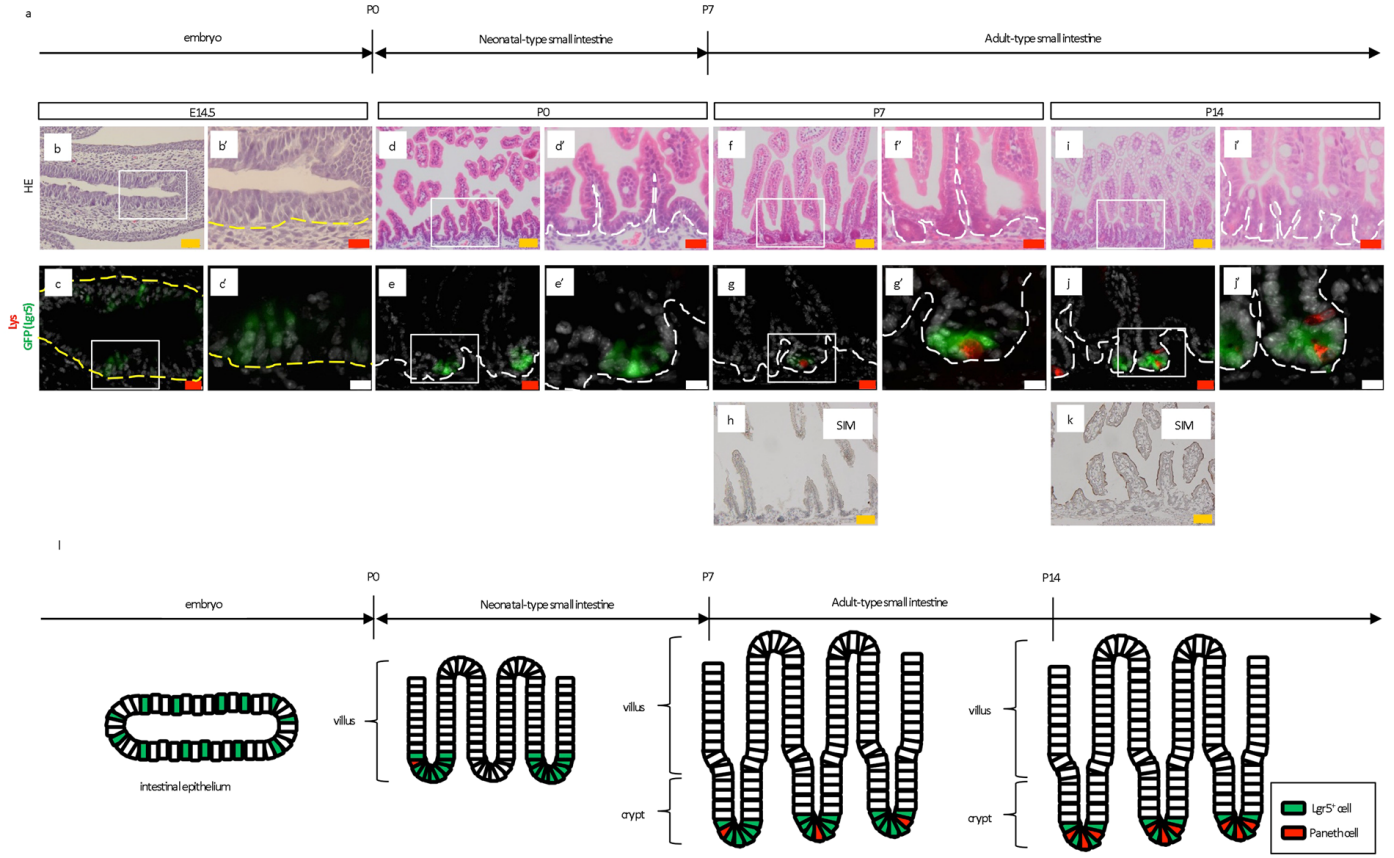
Maturation of neonatal small intestine to possess features comparable to adult intestine. (**a**) Developmental process of normal small intestine. P; postnatal day. (**b**,**b’**,**d**,**d’**,**f**,**f’**,**i**,**i’**) H&E staining for small intestine at the indicated time points. (**c**,**c’**,**e**,**e’**,**g**,**g’**,**j**,**j’**) Immunostaining for lysozyme (red, Lys) on sections prepared from *Lgr5* ^*EGFP*-*IRES*-*CreERT2*/+^ mice at the indicated time points (green, Lgr5). Higher magnification images (**b’**,**c’**,**d’**,**e’**,**f’**,**g’**,**i’**,**j’**) of the correspondent boxed area are shown on the right or lower side of the respective images. (**h**,**k**) Immunostaining of intestinal tissues revealing maturation into sucrose-isomaltase (SIM)-expressing brush border cells. Yellow and white dashed lines indicate the boundary between the epithelium and mesenchyme or lamina propria, respectively. Scale bars: orange; 50 μm; red, 20 μm; white, 10 μm. E; embryonic day. P; postnatal day. (**l**) Graphical explanation of the developmental process observed in embryonic, neonatal, and adult small intestine from *Lgr5* ^*EGFP*-*IRES*-*CreERT2*/+^ mice. P; postnatal day.

To confirm and precisely depict the process of small intestine and colon maturation from embryonic day 14.5 (E14.5) to postnatal day 14 (P14), *Lgr5* ^*EGFP*-*IRES*-*CreERT2*/+^ mice^6^ were examined at the respective ages in days (Fig. 1a). Consistent with previous findings^7^, histological analysis demonstrated crypts in the P5 small intestine (Supplementary Fig. 1i,i’). These crypts were not clearly detected at E14.5 (Fig. 1b,b’), P0 (Fig. 1d,d’), P1 (Supplementary Fig. 1a,a’), or P3 (Supplementary Fig. 1e,e’). Moreover, crypt length was found to increase from P5 (Supplementary Fig. 1i,i’) to P14 (Fig. 1f,f’, Supplementary Fig. 1m,m’, Fig. 1i,i’), where a significant difference was observed between P5 and P14 (Supplementary Fig. 2a). Villi—which reportedly begin to develop at E15^8^—had already formed at P0 (Fig. 1d,d’) and showed a significant difference in length from that observed at P14 (Supplementary Fig. 2b). In agreement with the previously reported emergence of immature Paneth cells at P5^7^, immunostaining for lysozyme demonstrated the appearance of approximately one Paneth cell per crypt at around P5 (Supplementary Fig. 1j,j’). Paneth cells were rarely observed in the crypts at earlier stages (Fig. 1c,c’,e,e’; Supplementary Fig. 1b,b’,f,f’). Furthermore, Lgr5 expression was monitored by GFP fluorescence, which showed that Lgr5^+^ cells localized to the inter-villus at E14.5 (Fig. 1c,c’), as well as P0, P1, and P3 (Fig. 1e,e’; Supplementary Figs 1b,b’,f,f’). Subsequently, Lgr5^+^ cells appeared in the crypts at P5, and the number per crypt significantly increased at P14 compared with P5 (Fig. 1g,g’; Supplementary Fig. 1j,j’,n,n’, Fig. 1j,j’, Supplementary Fig. 2c)^7, 9, 10^. In a single crypt of P14 mice, multiple Paneth cells alternated with Lgr5^+^ cells (Fig. 1j,j’) in accordance with the morphology of mature crypts in adult mice^4^. Neonatal small intestine differentiation analyses revealed PAS-stained goblet cells and alkaline phosphatase (ALP)^+^ enterocytes at P1 (Supplementary Fig. 1c,d,g,h,k,l,o,p) and villus sucrose-isomaltase (SIM)—a marker for mature brush border—starting at P10, which later expanded throughout the entire villus (Fig. 1h; Supplementary Fig. 1q,q’; Fig. 1k).

**Figure 2:**
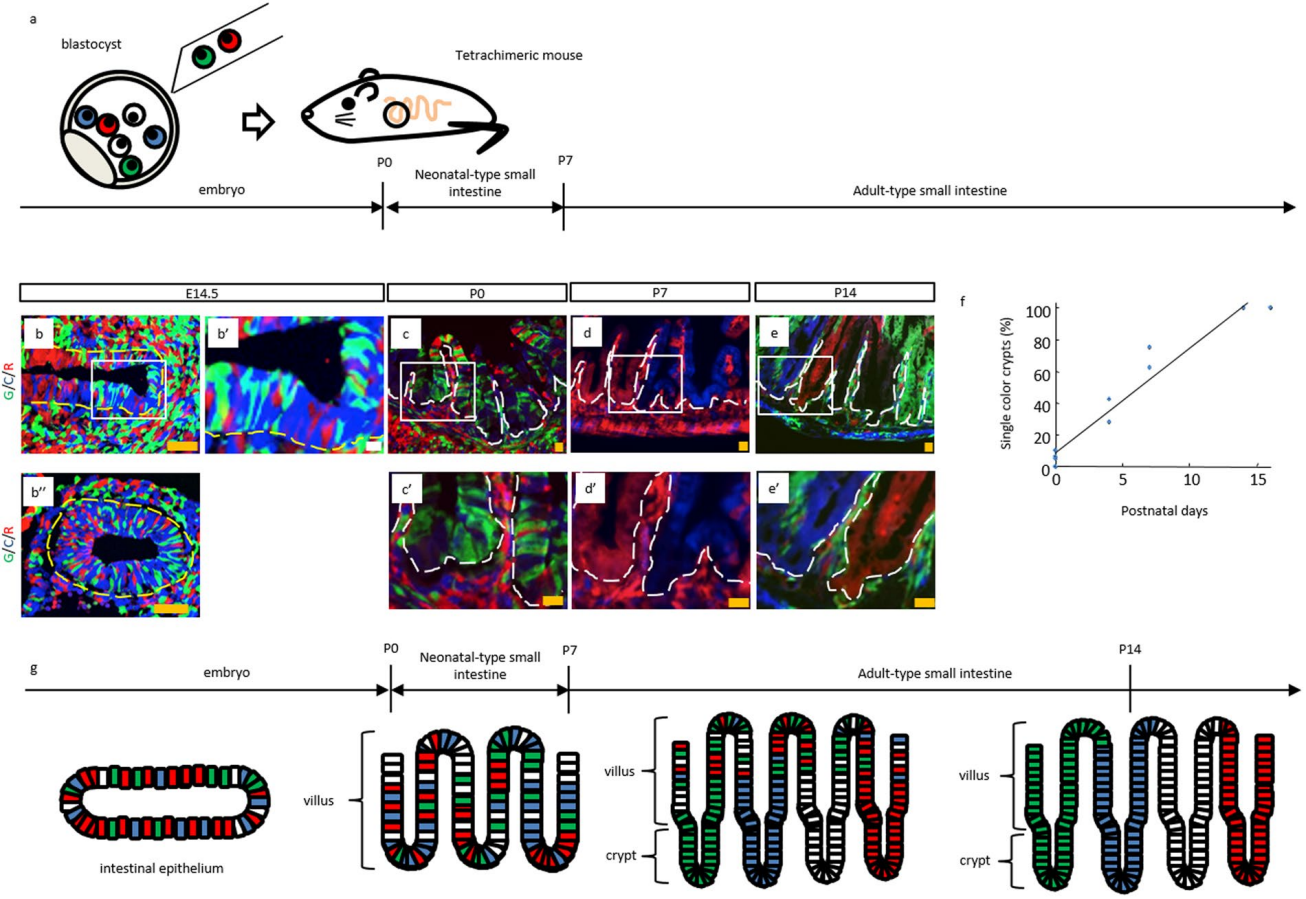
Change in cellular pattern of crypts and villi analyzed in tetrachimeric mice. (**a**) Schematic representation of production and intestinal development of tetrachimeric mice. (**b**,**b’**,**b”**,**c**,**c’**,**d**,**d’**,**e**,**e’**) Representative fluorescent images of tetrachimeric mice at the indicated time points. Higher magnification images (**b’**,**c’**,**d’**,**e’**) of the corresponding boxed area are shown on the right or lower side of the respective images. G, C, R; GFP, CFP, RFP, respectively. Yellow and white dashed lines indicate the boundary between the epithelium and mesenchyme or lamina propria, respectively. Scale bars: orange, 50 μm; white, 10 μm. E; embryonic day. P; postnatal day. (**f**) Percentage of single color crypts. N = 5 (P0); n = 2 (P4); n = 2 (P7); n = 2 (P14); n = 2 (P16). (**g**) Graphical representation of the developmental process observed in embryonic, neonatal, and adult small intestine from tetrachimeric mice. P; postnatal day.

Next, clonality of the neonatal small intestine was assessed. To this end, previously described tetrachimeric mice^11^ were analyzed, which is generated by injecting three kinds of embryonic stem (ES) cells in which the Rosa26 locus was knocked-in by EGFP, CFP, and mRFP1 into uncolored blastocysts (Fig. 2a). A polyclonal intestinal epithelium was observed at E14.5 (Fig. 2b,b”); however, the vertical axis of crypt-villus unit in the tetrachimeric mice was composed of single-colored cells at P14 (Fig. 2e,e’), demonstrating that the cells of each crypt-villus unit at P14 originated from a single stem cell located in the crypt base^6, 12^. This result corresponds to the previous study reporting the change in the cellular pattern of villi^13^; from a mottled pattern at P0 (Fig. 2c,c’; Supplementary Fig. 3a,b) to a striped pattern at P14 (Fig. 2e,e’; Supplementary Fig. 3f–h). In addition, the ratio of single color crypts was shown to increase from P0, such that all the crypts observed were composed of cells with a single color around P15 (Fig. 2c,c’,d,d’,e,e’,f; Supplementary Fig. 3). This result suggested that the crypt clonality shifted from polyclonal to monoclonal, consistent with a previous report^13^. Furthermore, analysis at P7 revealed the coexistence of monoclonal crypts and villi with a mottled cellular pattern (Fig. 2d,d’; Supplementary Fig. 3c–e), indicating that self-renewal and differentiation of stem cells started around P7. Based on the appearance of crypt-like structures in the small intestine at P7^14^ (Fig. 1f,f’), which are indicative of clonal expansion of stem cells as well as the differentiation of cells (Fig. 2d,d’), the small intestines of mice younger than P7 were described as neonatal-type and those in mice older than P7 as adult-type (Figs 1l and 2g).

For the proximal colon, histological analysis revealed mucosal folds at P0 (Supplementary Fig. 4a,a’), invagination of the mucosal folds into the lamina propria until P5 (Supplementary Fig. 4d,d’,g,g’), and immature crypts at P7 (Supplementary Fig. 4j,j’). The crypts then exhibited matured and longer structures formed at P10 and P14 (Supplementary Fig. 4m,m’,p,p’), where a significant difference was observed between P5 and P14 (Supplementary Fig. 4s)^15^. The developing colons were also classified into immature neonatal-type and adult-type based on crypt formation around P7. Notably, both Lgr5^+^ cells (Supplementary Fig. 4b,e,h,k,n,q) and PAS-stained goblet cells (Supplementary Fig. 4c,f,i,l,o,r) were detected throughout development from P0 to P14; however, no significant differences in Lgr5^+^ cells per crypt were observed between P0 and P14 (Supplementary Fig. 4t). Consistent with previous reports^7–9, 13–15^, our findings demonstrate the dynamic changes in the structure of the small intestine (Fig. 1l) and the colon (Supplementary Fig. 7a) during the two weeks after birth. In the subsequent transplantation experiments, the indicators described above were further analyzed to determine whether maturation proceeded in a normal manner.

### Establishment of a murine allotransplantation system of intestinal tissues into the subrenal capsule that recapitulates normal development

**Figure 3:**
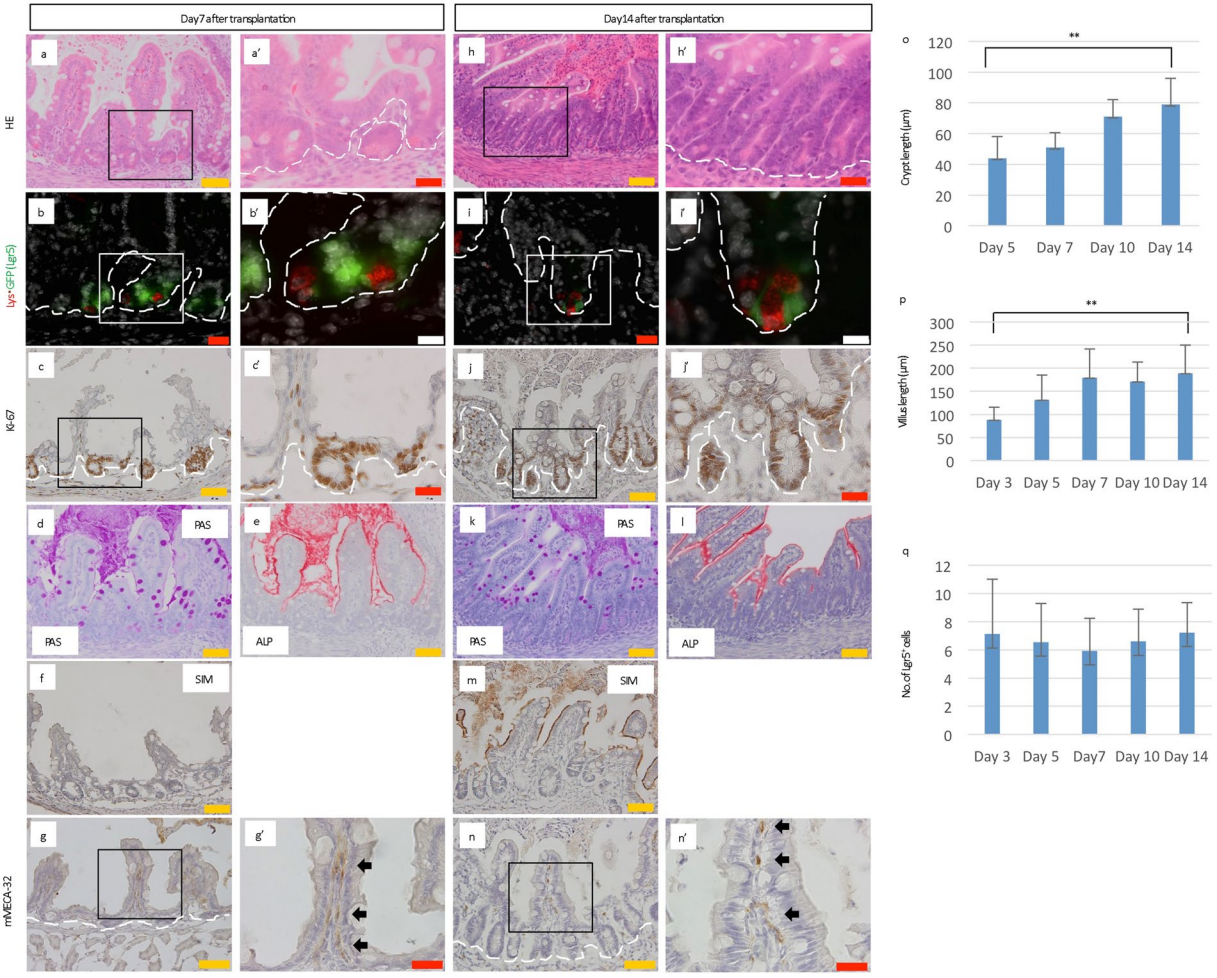
The subrenal capsule can support the development of the transplanted neonatal small intestine to complete maturation. (**a**,**a’**,**h**,**h’**) H&E staining for transplanted small intestine harvested from P0 *Lgr5* ^*EGFP*-*IRES*-*CreERT2*/+^ mice at the indicated days post-transplantation. (**b**,**b’**,**i**,**i’**) Immunostaining for lysozyme (red, Lys) on sections of transplanted small intestine derived from P0 *Lgr5* ^*EGFP*-*IRES*-*CreERT2*/+^ mice at the indicated days after transplantation (green, Lgr5). (**c**,**c’**,**j**,**j’**) Immunostaining for Ki-67 (brown), as a marker for cell proliferation, on sections of transplanted small intestine derived from P0 *Lgr5* ^*EGFP*-*IRES*-*CreERT2*/+^ mice at the indicated days post-transplantation. (**d**,**k**) Periodic acid-Schiff (PAS) staining for transplanted small intestine harvested from P0 *Lgr5* ^*EGFP*-*IRES*-*CreERT2*/+^ mice at the indicated days after transplantation. (**e**,**l**) Alkaline phosphatase staining of transplanted small intestine harvested from P0 *Lgr5* ^*EGFP*-*IRES*-*CreERT2*/+^ mice at the indicated days after transplantation. (**f**,**m**) Immunostaining of transplanted intestinal tissue revealing cell maturation based on sucrose-isomaltase (SIM) expression. (**g**,**g’**,**n**,**n’**) Immunostaining for mMECA-32 (brown) (**g’**,**n’**: black arrows), as a marker of vasculature ingrowth, on sections of transplanted small intestine derived from P0 *Lgr5* ^*EGFP*-*IRES*-*CreERT2*/+^ mice at the indicated days after transplantation. Higher magnification images (**a’**,**b’**,**c’**,**g’**,**h’**,**i’**,**j’**,**n’**) of the corresponding boxed area are shown on the right side of the respective images. White dashed lines indicate the boundary between the epithelium and lamina propria. Scale bars: orange, 50 μm; red, 20 μm; white, 10 μm. (**o**) The crypt length of transplanted small intestine harvested from P0 *Lgr5* ^*EGFP*-*IRES*-*CreERT2*/+^ mice at the indicated days after transplantation. Error bars indicate standard deviation. **p < 0.001. D; Days after transplantation. The numbers of crypts analyzed were as follows: D5; n = 62 in 10 transplants, D7; n = 47 in 8 transplants, D10; n = 50 in 7 transplants, and D14; n = 57 in 11 transplants derived from 4 mice (D5), 2 mice (D7), 2 mice (D10), and 3 mice (D14), respectively. (**p**) The villus length of transplanted small intestine harvested from P0 *Lgr5* ^*EGFP*-*IRES*-*CreERT2*/+^ mice at the indicated days after transplantation. Error bars indicate standard deviation. **p < 0.001. D; Days after transplantation. The number of villi analyzed were as follows: D3; n = 48 in 8 transplants, D5; n = 66 in 10 transplants, D7; n = 44 in 8 transplants, D10; n = 50 in 7 transplants, D14; n = 49 in 11 transplants derived from 2 mice (D3), 4 mice (D5), 2 mice (D7), 2 mice (D10), and 3 mice (D14), respectively. (**q**) The number of Lgr5-EGFP^+^ cells per inter-villus region or crypt. Error bars indicate standard deviation. D; Days after transplantation. The numbers of crypts analyzed were as follows: D3; n = 55 in 8 transplants, D5; n = 40 in 10 transplants, D7; n = 41 in 8 transplants, D10; n = 45 in 7 transplants, and D14; n = 40 in 11 transplants derived from 2 mice (D3), 4 mice (D5), 2 mice (D7), 2 mice (D10), and 3 mice (D14), respectively.

Next, to investigate whether neonatal intestines can mature without food intake, P0 intestines were transplanted into a microenvironment sequestered from the effects of digestion (Supplementary Figs 5, 6a–f and 7b). Small intestine and proximal colon tissues were dissected from P0 *Lgr5* ^*EGFP*-*IRES*-*CreERT2*/+^ mice and 2 mm-long pieces of the tissues were transplanted into the subrenal capsules of 8-week-old *C57BL*/*6 J* mice^16^ (Supplementary Fig. 6a–f). The mice were subsequently fed a normal diet without additional treatment. Gross observation of the transplanted small intestinal tissues at 14 days after transplantation indicated that the transplanted tissues grew to form normal tissue structures (Fig. 3h,h’; Supplementary Fig. 8o,o’) comparable with those of P14 mice (Fig. 1i,i’; Supplementary Fig. 4p,p’). In addition, crypt length increased from day 5 (Fig. 3a,a’,h,h’; Supplementary Fig. 8h,h’,o,o’), resulting in significantly longer crypts at day 14 than day 5 (Fig. 3o). The villi also started to grow longer from day 3 (Fig. 3a,a’,h,h’; Supplementary Fig. 8a,a’,h,h’,o,o’), and were significantly longer by day 14 (Fig. 3p). A more detailed analysis revealed that Lgr5^+^ cells appeared at day 3 (Supplementary Fig. 8b,b’), whereas Paneth cells were observed from day 5 (Fig. 3b,b’; Supplementary Fig. 8i,i’,p,p’). In addition, the crypts of the transplanted small intestines at day 14 were characterized by alternating Lgr5^+^ cells and lysozyme-expressing Paneth cells (Fig. 3i,i’). Although no significant difference in number of Lgr5^+^ cells per crypt was observed between days 3 and 14 (Fig. 3q), Ki-67^+^ cells were observed not only at the crypt base but also in the whole crypt, suggestive of healthy status of transplanted intestines to some extent (Fig. 3c,c’,j,j’, Supplementary Fig. 8c,j,q). We also examined whether the transplanted small intestine underwent functional maturation. From day 3 post-transplantation, both PAS-stained goblet cells (Fig. 3d,k; Supplementary Fig. 8d,k,r) and ALP^+^ enterocyte were detected (Fig. 3e,l; Supplementary Fig. 8e,l,s), whereas overall villus were confirmed to express SIM at day 10 (Fig. 3f,m; Supplementary Fig. 8f,m,t). These results indicated that all components of normal small intestines had been produced from transplanted P0 small intestine. Furthermore, immunostaining for mouse-specific pan-endothelial cell antigen (mMECA-32) revealed vascular ingrowths into transplanted intestines, which could support the self-renewal and differentiation of Lgr5^+^ stem cells (Fig. 3g,g’,n,n’; Supplementary Fig. 8g,n,u).

The transplanted colon at day 14 also emulated native tissues: mucosal folds were shorter (Supplementary Fig. 9u,u’) compared with those at day 3 (Supplementary Fig. 9a,a’). In addition, there was significant difference in crypt length between days 5 and 14 (Supplementary Fig. 10a), and both Lgr5^+^ cells (Supplementary Fig. 9v) and goblet cells (Supplementary Fig. 9x) were present at least until day 14. From days 3 to 10, transplanted colons (Supplementary Fig. 9a–t) exhibited histology similar to those of their counterpart tissues that matured under normal developmental conditions (Supplementary Fig. 4d–o). Lgr5^+^ cells were detected from day 3 (Supplementary Fig. 9b,g,l,q,v), although no significant differences in the number of Lgr5^+^ cells per crypt were observed between P3 and P14 (Supplementary Fig. 10b), consistent with physiological conditions (Supplementary Fig. 4t). Moreover, both Ki-67-positive proliferating crypt cells (Supplementary Fig. 9c,h,m,r,w) and mMECA-32-positive innervated vascular cells were observed (Supplementary Fig. 9e,j,o,t,y), as well as transplanted small intestines. These observations suggest that, even in the absence of digestion, neonatal murine small intestine and colon tissues develop normally, yielding all the expected components in the correct configuration (Supplementary Figs. 5 and 7b).

### Multicolor lineage-tracing of Lgr5^+^ stem cells in transplanted intestines reveals autonomous construction of mature, stem cell-derived tissues

**Figure 4:**
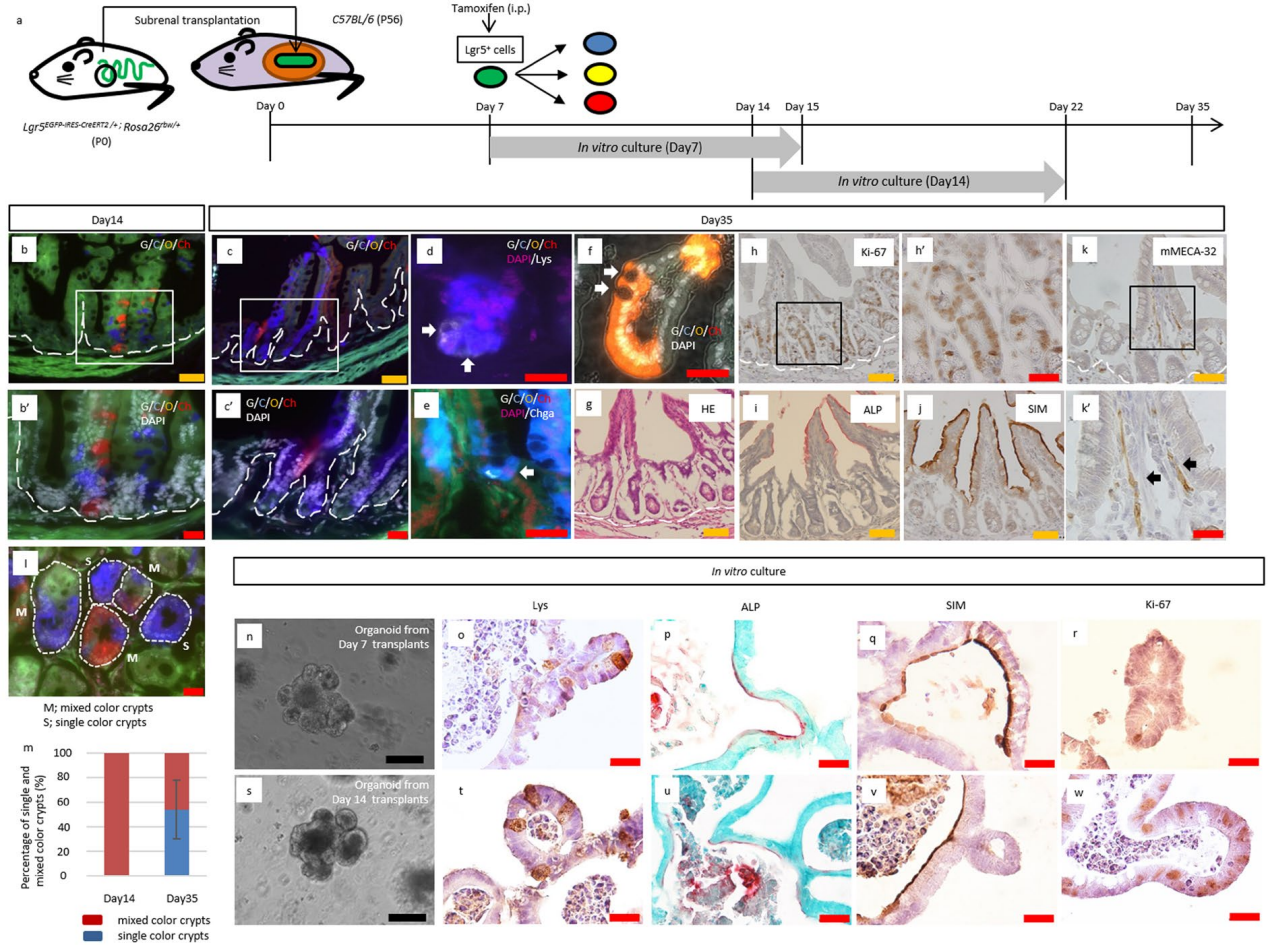
Multicolor lineage tracing of Lgr5^+^ cells revealed that the intestinal stem cells at P7 can autonomously differentiate into all types of cells that configure the crypt-villus unit. (**a**) Schematic protocol of transplanting neonatal intestine into subrenal capsule, followed by tamoxifen induction at 7 days after transplantation and harvest at 14 days or 35 days after transplantation. To investigate whether transplanted proximal regions of small intestines contained crypts with differentiation and proliferation capacities, crypts were isolated from grafts harvested at 7 or 14 days after transplantation and cultured *in vitro* for 8 days, at which point the resulting organoids were analyzed. P; postnatal day. (**b**,**b’**,**c**,**c’**) Fluorescent images of transplanted small intestines, in which multicolor labelling was induced in Lgr5^+^ cells, followed by lineage tracing until day 14 (**b**) or 35 (**c**). The number of transplants analyzed were as follows: Day 14, n = 4; Day 35, n = 8. Transplanted intestines were derived from 2 mice (D14) and 4 mice (D35), respectively. (**d**, **e**) Co-staining of the clones with the marker of differentiated lineages; Paneth cells (Lys; Lysozyme) (**d**), enteroendocrine cells (Chga; Chromogranin A) (**e**) (**d**,**e**: white arrows). (**f**) Representative image of Lgr5^+^ cell-derived clones that contained goblet cells, in which apical localization of secretory granules and compressed nuclei toward basal direction were observed (white arrows). (**g**) H&E staining for the transplanted small intestine harvested from P0 *Lgr5* ^*EGFP*-*IRES*-*CreERT2*/+^; *Rosa26* ^*rbw*/+^ mice at day35 after transplantation. (**h**,**h’**) Immunostaining for Ki-67 (brown), as a marker for cell proliferation, on sections of the transplanted small intestine derived from P0 *Lgr5* ^*EGFP*-*IRES*-*CreERT2*/+^; *Rosa26* ^*rbw*/+^ mice. (**i**) Alkaline phosphatase staining for the transplanted small intestine harvested from P0 *Lgr5* ^*EGFP*-*IRES*-*CreERT2*/+^; *Rosa26* ^*rbw*/+^ mice. (**j**) Immunostaining of transplanted intestinal tissue revealing maturation with the brush border cell marker sucrose-isomaltase (SIM). (**k**,**k”**) Immunostaining for mMECA-32 (brown) (**k’**: black arrows), as a marker of vasculature ingrowth, on sections of transplanted small intestine derived from P0 *Lgr5* ^*EGFP*-*IRES*-*CreERT2*/+^; *Rosa26* ^*rbw*/+^ mice. Higher magnification images (**b’**,**c’**,**h’**,**k’**) of the corresponding boxed area are shown below or on the right of the respective images. (**l**) Representative image of horizontal section of transplanted small intestine. Day 35 grafts were analyzed. M; mixed color crypts, S; single color crypts. (**m**) The percentage of single and mixed color crypts. Day 14, 80 crypts in 4 transplants were assessed; Day 35, 50 crypts in 8 transplants. Error bars indicate standard deviation. (**n–w**) Grafts were harvested at 7 (**n–r**) or 14 days (**s–w**) after transplantation and crypts were isolated from them to generate organoids for analysis at day 8. (**n**,**s**) Representative images of organoids with crypt-like structures penetrating outside. This feature rather than spherical shape demonstrated that the organoids were derived from matured crypts. (**o**,**t**) Representative images of immunostaining of organoids for Lys. Brown-stained cells indicate Lys^+^ Paneth cells. (**p**,**u**) Representative images of alkaline phosphatase staining of organoids. Red-stained brush border enzyme indicated that cultured organoids had expanded with differentiating into matured villi. Nuclei were stained with methyl-green. (**q**,**v**) Immunostaining of organoid revealing maturation into the cells that expressed brush border enzyme, sucrose-isomaltase (SIM). (**r**,**w**) Immunostaining for Ki-67 (brown) as a marker for cell proliferation. (**o**–**r**,**t**–**w**) Lower magnification images of the corresponding images are shown in Supplementary Fig. 13h–k, and l–o, respectively. White dashed lines indicate the boundary between epithelium and lamina propria. Scale bars: black, 100 μm; orange, 50 μm; red, 20 μm.

To test whether stem cell activity enables neonatal intestine development independently from food intake and subsequent stimulus by digestion, multicolor lineage tracing of Lgr5^+^ stem cells was carried out in the transplanted neonatal intestine (Fig. 4a; Supplementary Fig. 12a). Small intestine and colon tissues were collected from P0 *Lgr5* ^*EGFP*-*IRES*-*CreERT2*/+^; *Rosa26* ^*rbw*/+^ ^17, 18^ mice and transplanted into 8-week-old *C57BL*/*6* mice (Fig. 4a; Supplementary Figs 6, 12a). By means of intraperitoneal injection of tamoxifen into the host mice, CreERT2-mediated recombination followed by random expression of mCerulean, mCherry, or mOrange was induced in Lgr5-expressing stem cells of the transplanted neonatal intestine (Fig. 4a; Supplementary Fig. 12a). The crypt structure was formed before P7 in both native tissues (Fig. 1f,f’; Supplementary Fig. 4j,j’) and transplanted intestines (Fig. 3a,a’; Supplementary Fig. 9k,k’). The tetrachimeric mice were furthermore shown to develop monoclonal crypts in the small intestine at P7 (Fig. 2d,d’), suggesting that the adult epithelium, which possesses a cell renewal system, emerges around P7. Accordingly, it was hypothesized that Lgr5^+^ stem cells in the transplanted tissues at day 7 can both supply stem cells and differentiate into several types of cells, which support continual turnover^19^. To investigate this hypothesis, Lgr5^+^ cells were labeled at 7 days after transplantation of P0 intestines and their lineage was traced (Fig. 4a; Supplementary Fig. 12a). Analysis of the transplanted intestines at 7 days after tamoxifen induction revealed that the crypts contained colored cells, demonstrating the presence of descendants of Lgr5^+^ stem cells (Fig. 4b,b’; Supplementary Figs 11, 12b,b’,k). The presence of a patch of cells, which expressed the same single color, further indicated the presence of cells of a single clone derived from a single Lgr5^+^ stem cell. Several such patches in a single crypt thus indicated that multiple stem cells had been present, as previously reported for normal intestinal tissue^13^. In contrast, at 28 days after tamoxifen induction, crypt-villus units in the small intestine (Fig. 4c,c’; Supplementary Fig. 11) and crypts in the colon (Supplementary Fig. 12c,c’,k) were shown to be composed of cells of a single color. Quantification of single color crypts demonstrated that about 50% of crypts in the transplanted small intestine and colon were monoclonal 28 days after tamoxifen induction (Fig. 4l,m; Supplementary Fig. 12i,j). This ribbon-like arrangement of cells from the stem cell-containing crypt base to several types of differentiated cells suggests that single stem cells had continuously supplied these cells by self-renewal and the subsequent production of transit-amplifying cells, thereby substituting other stem cells and their descendants, as observed at day 7 (Fig. 4b,b’; Supplementary Fig. 12b,b’). After a 4-week-long chase after tamoxifen induction (D35), not all crypts had become monoclonal, consistent with previous reports^12, 20^.

In addition to the analysis of transplanted P0 intestines derived from *Lgr5* ^*EGFP*-*IRES*-*CreERT2*/+^ mice using cell-fate mapping with tamoxifen induction (Fig. 4a-c’; Supplementary Fig. 12a-c’), we also examined whether functional maturation proceeded in transplanted tissues. Notably, lysozyme-expressing Paneth cells were detected in transplanted small intestines at day 35 (Fig. 4d), and transplanted small intestine and colon were both confirmed to differentiate into all lineages—including ChromograninA (Chga)^+^ enteroendocrine cells (Fig. 4e; Supplementary Fig. 12d), goblet cells (Fig. 4f; Supplementary Fig. 12e), enterocytes (Fig. 4i), and SIM-expressing mature villi (Fig. 4j). Gross observation of the transplanted intestinal tissues 35 days after transplantation indicated that the tissues grew to form normal structures (Fig. 4g; Supplementary Fig. 12f). Moreover, proliferating crypt cells (Fig. 4h,h’; Supplementary Fig. 12g,g’) and vascular ingrowth into the transplanted tissues were also detected (Fig. 4k,k’; Supplementary Fig. 12h,h’). These findings support that neonatal intestinal stem cells can differentiate into all types of cells that constitute the crypt-villus unit of the small intestine (Supplementary Fig. 11) or all the cells of the crypts in the colon (Supplementary Fig. 12k), despite not being located in the native position where they would usually be exposed to various nutrients and effects of digestive activity.

### Organoid culture of transplanted small intestine crypts demonstrates that the subrenal capsule is sufficient for neonatal intestinal maturation

Our immunohistochemical analysis and lineage-tracing results demonstrated the functional maturation of transplanted small intestine into several types of differentiated cells and formed healthy crypts containing Lgr5^+^ stem cells. Thus, we sought to prove the considerable maturation of transplanted small intestine by *in vitro* culture of crypts harvested from the grafts. Human and mouse fetal intestines contain proliferative and immature progenitors that can be expanded *in vitro* as fetal enterospheres (FEnS)^21^, which specifically develop from the proximal regions of neonatal epithelial cell cultures^21^. Another study demonstrated that an abundance of these spheroid-generating cells decreased with maturation, whereas the proportion of organoids with crypt-like structures penetrating toward the outside increases accordingly^22^. Therefore, we analyzed transplanted proximal regions of small intestine in P0 mice by analyzing grafts harvested at days 7 or 14. Consistent with previous findings, FEnS were generated (92.6%) from epithelial cells of proximal small intestines at P0 (Supplementary Fig. 13a,b,f), while crypts from P7, P14, and P56 mice formed organoids (Supplementary Fig. 13a,c–e). Notably, the proportion of organoid-forming crypts was higher than P0; with 84.4% and 100% of crypts of P7 and P14 mice, respectively (Supplementary 13f). Similar to physiological tissues, transplanted P0 intestines at days 7 or 14 after grafting also contained crypts that could form organoids (99.4%, and 100%, respectively; Fig. 4n,s; Supplementary Fig. 13g). Moreover, organoids generated from grafted crypts differentiated into Paneth cells (Fig. 4o,t; Supplementary Fig. 13h,h’,l,l’), enterocytes (Fig. 4p,u; Supplementary Fig. 13i,m), SIM-expressing matured villi (Fig. 4q,v; Supplementary Fig. 13j,n), and Ki-67^+^ cells (Fig. 4r,w; Supplementary Fig. 13k,o,o’), suggesting that under the subrenal capsule, transplanted neonatal intestine had matured while retaining its capacity for differentiation and proliferation. Thus, these findings led us to propose that the mechanism of structural development of neonatal intestinal tissue to maturity is not an extrinsic one, but rather an autonomous one.

## Discussion

In this study, an allotransplantation system of murine small intestine and colon tissues into the subrenal capsule was established, demonstrating that neonatal intestines give rise to mature and self-contained structures comparable with native tissues, even in the absence of the stimulus of food intake and digestive activities. Lineage-tracing of Lgr5^+^ stem cells in the transplanted intestine further revealed that this maturation process occurs independently of external stimuli and is underpinned by stem cell activity: stem cells in the transplanted intestines self-renew and produced differentiating cells that occupy the crypt-villus unit of the small intestine or the whole crypt of the colon. Of note, the matured intestines contained Paneth cells and deep crypt secretory cells—the niche cells for stem cells in the small intestine and the colon, respectively^4, 5^. These findings indicate that the autonomous environment for continual turnover was prepared, where the stem cells produced niche cells that supported the stem cells.

The period over which subrenal-transplanted intestinal tissues can be assessed may be limited by the accumulation of necrotic debris or digestive juice that cannot be excreted. When E14.5 rat small intestine is transplanted into the anterior chamber, it develops and differentiates to form standard small intestinal structures equipped with villi and crypts until 22 days after transplantation^2^. Although the subrenal capsule allows transplanted P4 small intestine to develop to form the crypts that contain Paneth cells until four weeks after transplantation, the intestine has been shown to degenerate at six weeks after transplantation^3^. Consistent with these previously reported observations in rats, degeneration of mucosal epithelia was observed at five weeks after transplantation in this study– normal structure of crypt or villi could not be observed although only goblet cells remained to be detected in transplanted small intestine and colon (data not shown), and the endpoint of the observation was thus set at five weeks after transplantation. Since tamoxifen was injected into the host mice seven days after transplantation of intestinal tissues, the fate of Lgr5^+^ stem cells in transplanted intestines was traced for four weeks. Considering that the turnover rate of intestines is less than two weeks^19^, this time period was considered sufficient for confirming the presence of a self-renewing system originating from stem cells.

The fact that intestinal organoids derived from human induced pluripotent stem (iPS) cells also develop normally when transplanted under the murine kidney capsule^16^ suggests that the development of intestines is similarly autonomous in mice and humans. In fact, the human intestine acquires mature characteristics during fetal life around 22 weeks of gestation, indicating that extrinsic factors—such as the role of microbiota, milk, or food intake—play a minor role in the process^23^. Our results suggest that this process is conserved in rodents in addition to the previous reports using rats^2, 3^; however the stages of crypt development occurs postnatally and in utero in mice and humans, respectively. The multicolor lineage-tracing system of transplanted tissues reported here facilitates the visualization of stem cell dynamics during neonatal development of intestines independent of digestive activity. This system would be useful for further investigations into the molecular mechanisms underlying intestinal maturation that seem to be independent from food intake. Other applications of this system may include investigations into the difference between embryonic/neonatal intestinal stem cells and adult stem cells, including whether the putative differences are accounted for by the autonomous regulation.

## Methods

### Animals

*C57BL*/*6J* and *Lgr5* ^*EGFP*-*IRES*-*CreERT2*/+^ ^6^ lines were purchased from Jackson Laboratories. *Rosa26* ^*rbw*/+^ lines^17, 18^ and tetrachimeric mice^11^ were generated as previously described. Mice were bred and maintained at the Kansai Medical University Research Animal Facility in accordance with the Kansai Medical University guidelines. All animal experimental protocols were approved by the Kansai Medical University Welfare Committee.

### Tamoxifen induction

To induce CreERT2-mediated multi-color labeling in the transplanted intestine, host mice were intraperitoneally injected with Tamoxifen (Sigma) dissolved in corn oil (Sigma) at 7 days after transplantation and at a concentration of 7 mg per 40 g body weight.

### Subrenal capsule assay

P0 *Lgr5* ^*EGFP*-*IRES*-*CreERT2*/+^ and *Lgr5* ^*EGFP*-*IRES*-*CreERT2*/+^; *Rosa26* ^*CreERT2*/+^ mice were anesthetized with isoflurane, and 2 mm-long sections of the small intestine and proximal colon were collected right before transplantation and washed with cold phosphate-buffered saline (PBS). The harvested tissues were then transplanted under the kidney capsule as previously reported^16^. Briefly, 8-week-old recipient *C57*/*BL6* mice were anesthetized with an intraperitoneal injection of medetomidine-HCl (0.75 mg/kg; ZENOAQ), midazolam (4 mg/kg; SANDOZ), and butorphanol tartrate (5 mg/kg; Meiji), and the left side of the mouse was then prepped in sterile fashion. A small left-posterior subcostal incision was made to expose the kidney. A subcapsular pocket was created and the neonatal intestines were placed into the pocket. The kidney was then returned to the peritoneal cavity and mice were given an IP flush of Antisedan (0.75 mg/kg; ZENOAQ). Recipient mice were kept warm during and for one hour after surgery. Mice were then humanely euthanized at 3, 5, 7, 10, 14, and 35 days post-engraftment for further analysis.

### Organoid culture

Proximal regions of the small intestine were collected from P0, P7, P14, and P56 *C57BL*/*6 J* mice. Tissues were incised longitudinally and washed several times in cold PBS containing 500 µM dithiothreitol (DTT). The intestines were then cut into about 2 mm-long fragments in cold PBS and incubated in 10 mL of chelating buffer pH 7.3 (27 mM trisodium citrate, 5 mM Na_2_HPO_4_, 94 mM NaCl, 8 mM KH_2_PO_4_, 1.5 mM KCl, 0.5 mM DTT, 55 mM D-sorbitol, 44 mM sucrose) at 4 °C with constant stirring for 20 min^24^. The fragments were then transferred to 5 mL of fresh cold chelating buffer and vigorously shaken by hand (10 inversions). The chelating buffer was subsequently replaced with 5 mL of fresh cold chelating buffer and the procedure repeated 4–5 times to remove villi. The intestine fragments were incubated again in 10 mL of chelating buffer at 4 °C with constant stirring for 10 min, and the epithelium of neonatal P0 mice or crypts of P7, P14, or P56 mice were released into the chelating buffer by vigorous shaking by hand (10 inversions). The collected epithelial cells or crypts were cultured in Matrigel (Becton Dickinson Biosciences, San Jose, CA) in the presence of cytokines^25^. Briefly, epithelial cells or crypts were suspended in Matrigel (3–5 × 10^3^ cells/50 µL of Matrigel) and plated in 24-well plates (triplicate). Then, 0.75 mL of advanced DMED/F-12 medium supplemented with N-2, B-27, N-acetyl cysteine, Glutamax (Invitrogen), and cytokines (rmEGF: 50 ng/mL [Preprotech], rmNoggin: 100 ng/mL [Preprotech], rhR-spondin1-hFc: 1000 ng/mL) were added. The R-spondin1-hFc containing a C-terminal of human IgG was produced in our laboratory using cDNA kindly donated by Kyowa Hakko Kirin (Tokyo, Japan). Y-27632 (10 µM, Sigma) was also added to the culture medium. Every 2 or 4 days, all the culture medium in the wells was replaced with fresh medium. For transplanted proximal small intestinal tissues, tissues were harvested at 7 or 14 days after transplantation. Crypts were collected from grafts and used for *in vitro* culture according to the protocol described above.

### Histological analysis

Animals were anesthetized with isoflurane. Harvested tissues were processed to produce frozen or paraffin-embedded sections as previously reported^11, 26^. Hematoxylin and eosin (H&E), alkaline phosphatase, and Periodic acid-Schiff (PAS) staining were performed following a general protocol. For lineage-tracing analysis^17, 18, 27^, frozen tissues were cut into 7 μm slices. Traced cell lineage was analyzed using the fluorescent images of slices taken with an OLYMPUS BX63 (Olympus Corporation, Tokyo, Japan).

### Immunohistochemistry

Immunostaining for lysosome, Sucrase-Isomaltase, Ki-67, mMECA-32, and ChromograninA was performed on frozen sections using rabbit anti-lysozyme (A0099, DAKO, 1:1000), mouse anti-sucrase-isomaltase (sc-393470, clone: C-8, Santa Cruz, 1:50), rabbit anti-ki-67 (ab16667, clone: SP6, abcam, 1:500), rat anti-MECA-32 (NB100-77668, clone: MECA-32, NOVUS Biologicals, 1:50), and rabbit anti-ChoromograninA (ab15160, Abcam, 1:400) antibodies, respectively. Immunostaining for GFP to detect Lgr5 expression was performed on frozen sections or paraffin-embedded sections using rabbit anti-GFP (2956, clone: D5.1, Cell Signaling, 1:100) antibody. For DAB staining, slides were then incubated with amino acid polymer conjugated with peroxidase and Fab’ (Histofine simple stain rabbit MAX-PO (R), 414341, Nichirei Bioscience Inc.). Slides were visualized using metal-enhanced 3,3′- diaminobenzidine (DAB) and counterstained with hematoxylin. Images were acquired using an OLYMPUS BX41 microscope (OLYMPUS Corporation). When co-staining of Lys and GFP or co-staining with rainbow colors were performed, Alexa488-, Alexa594-, and Alexa750-conjugated secondary antibodies (1:200, Invitrogen) were used, followed by nuclei-staining using Hoechst33342 (Thermo Fisher Scientific, H3570). Fluorescent images were taken with an OLYMPUS BX63 microscope (Olympus Corporation, Tokyo, Japan).

### Statistical analyses

Crypt and villi lengths were measured using H&E-stained sections of normal small intestine and proximal colon, as well as transplanted small intestine and proximal colon. Lgr5-GFP^+^ cells were enumerated by observing immunostained sections. Analysis was performed using Student’s *t*-test.

### Ethics statement

All animal experiments were performed in accordance with the guidelines of Kansai Medical University and approved by the Kansai Medical University Animal Experiment Committee.

## Electronic supplementary material

Supplementary Information
