# Supplementary material for: Intestinal stem cells contribute to the maturation of the neonatal small intestine and colon independently of digestive activity
Source: Sci Rep. 2017 Aug 31;7:9891. doi: 10.1038/s41598-017-09927-2 (PMC5578958; doi:10.1038/s41598-017-09927-2)
Supplement: Supplementary file 1 — Supplementary Information [file 41598_2017_9927_MOESM1_ESM.pdf]

Intestinal stem cells contribute to neonatal small intestine and colon maturation independent of digestive activity

Hirotsugu Yanai[1,2], Naho Atsumi[1], Toshihiro Tanaka[1, 3], Naohiro Nakamura[1,3], Yoshihiro Komai[1,4], Taichi Omachi[1,5], Kiyomichi Tanaka[1], Kazuhiko Ishigaki[1], Kazuho Saiga[1], Haruyuki Ohsugi[1,4], Yoko Tokuyama[1], Yuki Imahashi[1], Shuichi Ohe[1, 6], Hiroko Hisha[1], Naoko Yoshida[1], Keiki Kumano[1], Masanori Kon[2] and Hiroo Ueno[1]*

[1]Department of Stem Cell Pathology, [2] Department of Surgery, [3]Third Department of Internal Medicine, [4]Department of Urology and Andrology and [5]Department of Pediatrics, [6]Department of Dermatology, Kansai Medical University, 2-5-1 Shin-machi, Hirakata, Osaka 573-1010, Japan

*Correspondence and requests for materials should be addressed to H.U. (hueno@hirakata.kmu.ac.jp).

Supplementary Figures

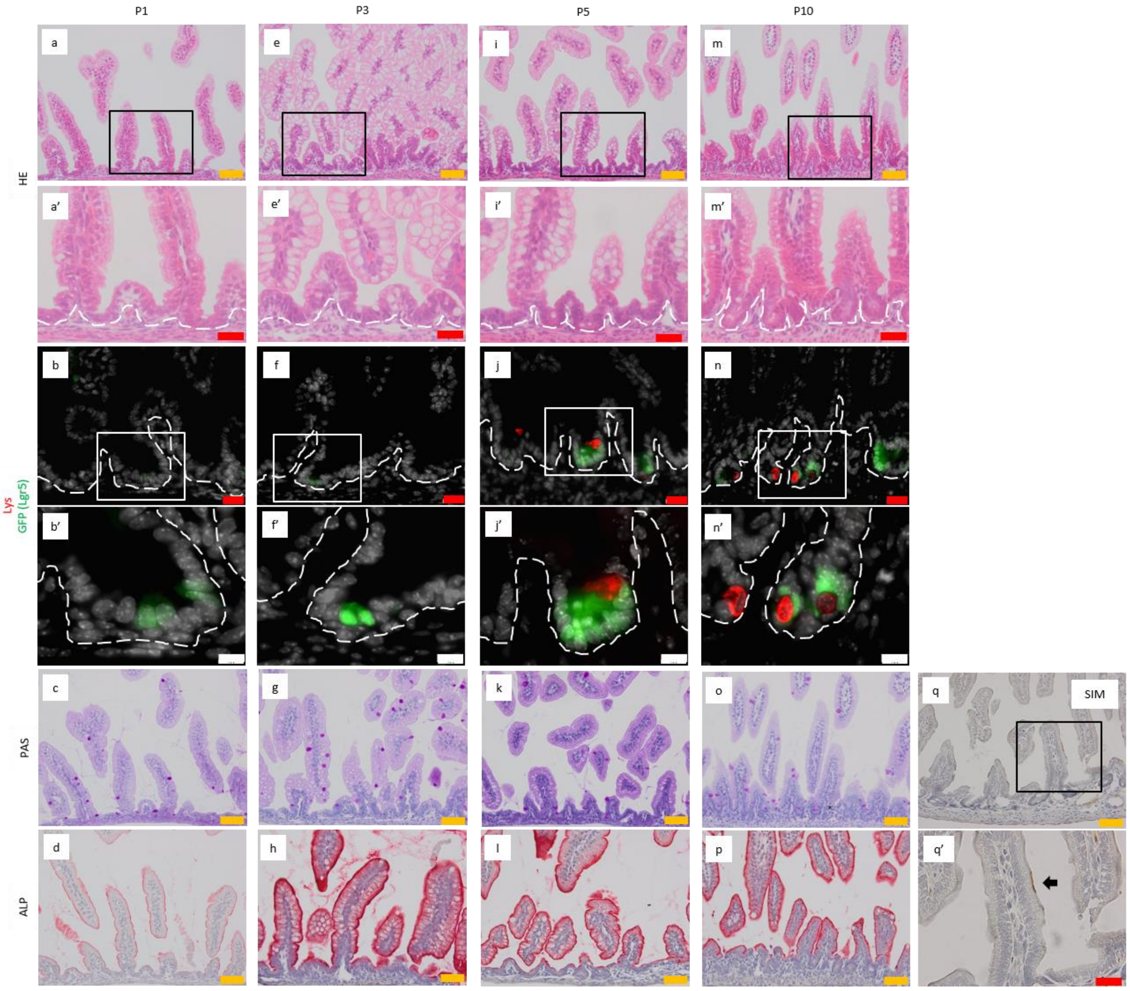

Supplementary Figure 1. Maturation of neonatal small intestine to possess features comparable to the adult intestine.

(a, a', e, e', i, i', m, m') H&E staining for small intestine at the indicated birth date.

(b, b', f, f', j, j', n, n') Immunostaining for lysozyme (red, Lys) on sections prepared from *Lgr5*$^{CreERT2/+}$ mice at the indicated birth date (green, Lgr5).

(c, g, k, o) Periodic acid-Schiff (PAS) staining for the small intestine at the indicated birth date.

(d, h, l, p) Alkaline phosphatase staining for the small intestine at the indicated birth date.

(q, q') Immunostatining of intestinal tissues for the brush border enzyme sucrose-isomaltase (SIM) (q'; arrow) was used to assess functional maturation.

Higher magnification images (a', b', e', f', i', j', m', n', q') of the corresponding boxed area were shown below the respective images. White dashed lines indicate the boundary between the epithelium and lamina propria. Scale bars: orange, 50 μm; red, 20 μm; white, 10 μm. P; postnatal day.

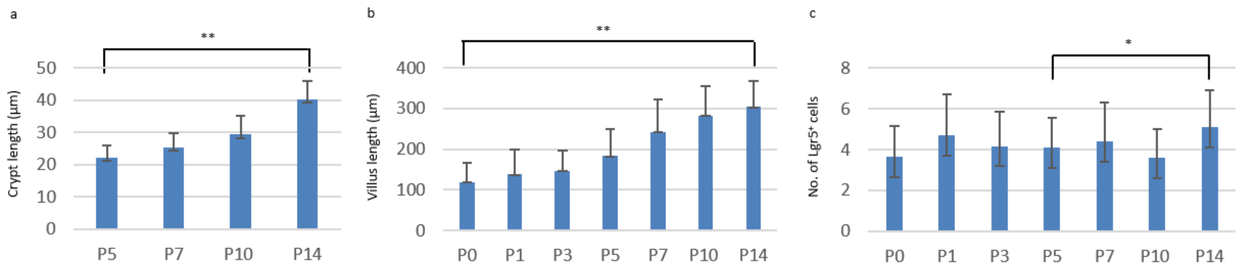

Supplementary Figure 2. Quantitative analysis of crypt length, villus length, and the number of Lgr5-EGFP^{+} cells in small intestine

(a) The crypt length of the small intestine of *Lgr5*$^{EGFP\text{-}IRES\text{-}CreERT2/+}$ mice at the indicated days. P; postnatal day. The number of mice analyzed were as follows; P5; n = 3, P7; n = 2, P10; n = 2, P14; n = 4. A total of 40 crypts at each age were assessed.

(b) The villus length of the small intestine of *Lgr5*$^{CreERT2/+}$ mice at the indicated days. P; postnatal day. The number of mice analyzed were as follows: P0; n = 4, P1; n = 4, P3; n = 3, P5; n = 3, P7; n = 2, P10; n = 2, P14; n = 4. From these mice, a total of 40 villi were assessed at each age.

(c) The number of Lgr5-EGFP^{+} cells per inter-villus resion (P0 to P3) or crypt (P5 to P14). P; postnatal day. The number of mice analyzed were as follows; P0; n = 4, P1; n = 4, P3; n = 3, P5; n = 3, P7; n = 2, P10; n = 2, P14; n = 4. A total of forty crypts were assessed at each age.

Error bars indicate standard deviation. **; $p < 0.001$, *; $p < 0.01$.

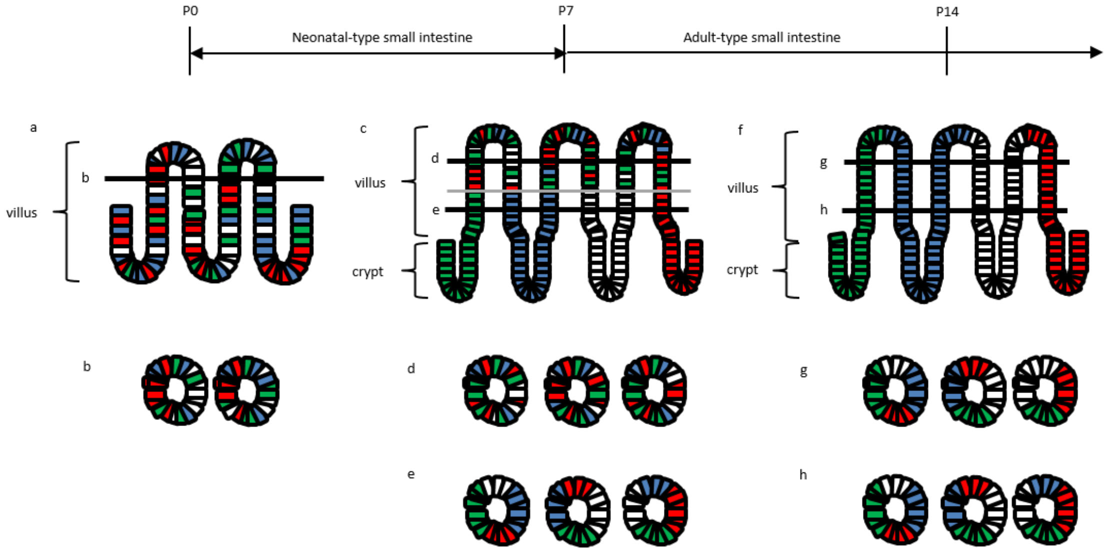

Supplementary Figure 3. The change in clonality of villi observed in tetrachimeric mice.

(a, b) Schematic representation of villi of small intestine at P0. Longitudinal section (a) and horizontal section of upper villi (a; black line) (b) are shown. A mingled pattern of four colors was observed in the horizontal section, suggesting that the villi are perfectly polyclonal.

(c-e) Schematic representation of villi of the small intestine at P7. Longitudinal (c) and horizontal sections of upper villi (c; upper black line) (d), and horizontal section of lower villi (c; lower black line) (e) are shown. The upper villi were yet to display a mingled pattern of four colors, whereas segmented single-colored areas were observed in the horizontal sections of the lower villi. Because each single-colored area can be considered derived from stem cells, it was suggested that P7 stem cells located at the crypt-base had started to supply their desendants to occupy the lower part of the villi. Multiple single-colored areas suggested that there existed several stem cells in the single crypt and therefore the lower villi were polyclonal. However, the difference in color arrangement was observed between the stem-cell derived multiple clones observed in lower villi and mingled pattern in upper villi. The gray line indicates the boundary between these two color patterns.

(f-h) Schematic representation of villi of small intestine at P14. Longitudinal section (f) and horizontal section of upper villi (f; upper black line) (g), and horizontal section of lower villi (f; lower black line) (h) are shown. In addition to lower villi (h), the horizontal section of upper villi (g) also showed segmented single-colored areas, suggesting that the stem cells had continuously supplied descendants up to the top of the villi.

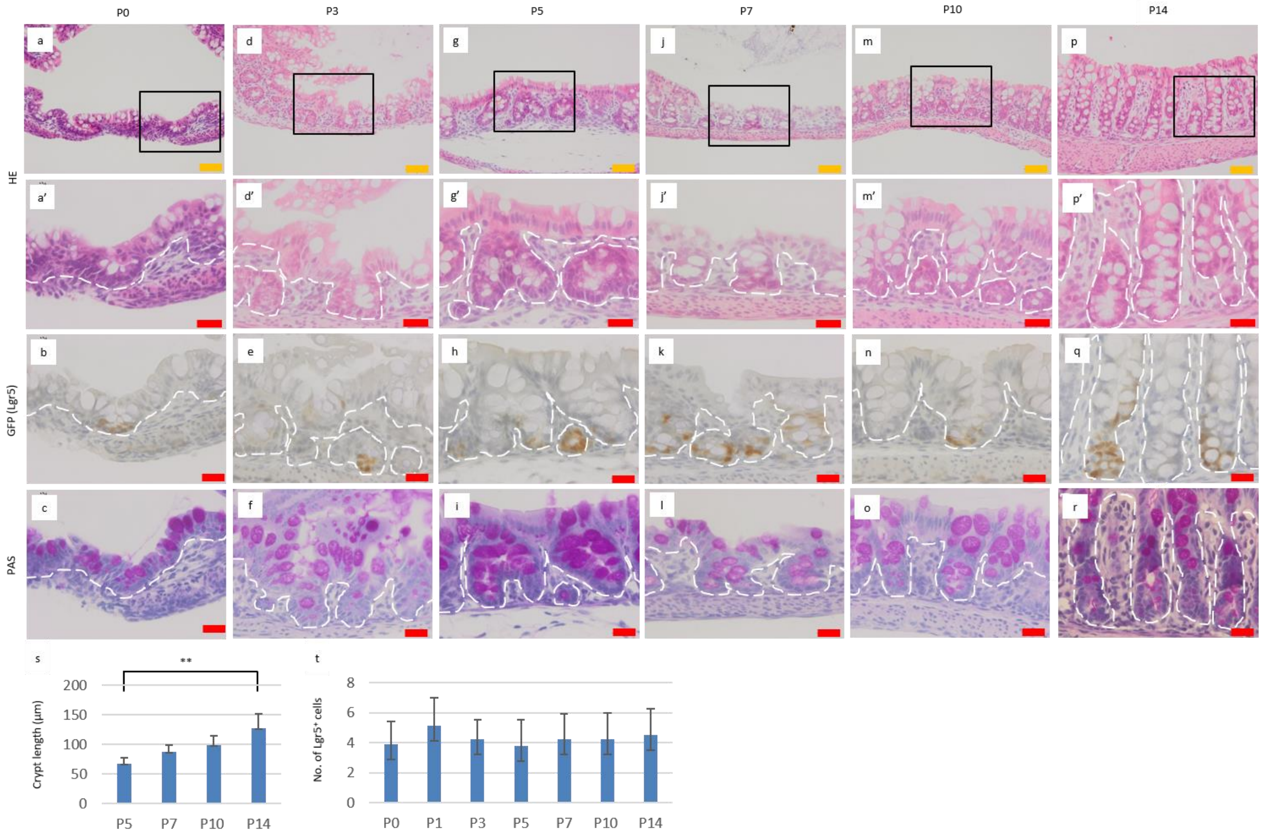

Supplementary Figure 4. Maturation of neonatal colon to possess features comparable to the adult intestine.

(a, a’, d, d’, g, g’, j, j’, m, m’, p, p’) H&E staining of the colon at the indicated birth date.

(b, e, h, k, n, q) Immunostaining of GFP (brown) to detect the expression of Lgr5 using the colon of $Lgr5^{EGFP\text{-}IRES\text{-}CreERT2/+}$ mice at the indicated birth date.

(c, f, i, l, o, r) Periodic acid-Schiff (PAS) staining of the colon at the indicated birth date.

Higher magnification images (a’, d’, g’, j’, m’, p’) of the corresponding boxed area are shown below the respective images. White dashed lines indicate the boundary between epithelium and lamina propria. Scale bars: orange, 50 μm; red, 20 μm; white, 10 μm. P; postnatal day.

(s) The crypt length of colon of $Lgr5^{EGFP\text{-}IRES\text{-}CreERT2/+}$ mice at the indicated days. Error bars indicate standard deviation. **; $p<0.001$. P; postnatal day. The number of mice analyzed were as follows: P5; n = 3, P7; n = 2, P10; n = 2, P14; n = 4. From these mice, 40 crypts in total at each age were assessed.

(t) The number of Lgr5-EGFP$^+$ cells per crypt. Error bars indicate standard deviation. P; postnatal day. The numbers of mice analyzed were as follows; P0; n = 3, P1; n = 4, P3; n = 3, P5; n = 3, P7; n = 2, P10; n = 2, P14; n = 4. From these mice, 40 crypts in total at each age were assessed.

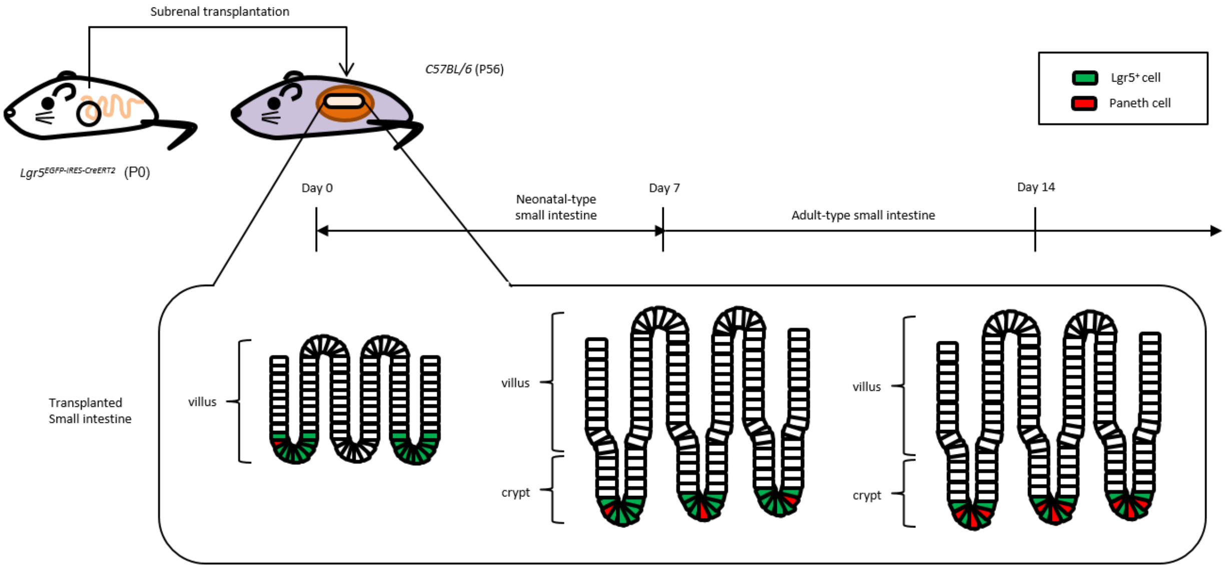

Supplementary Figure 5. Schematic representation: Establishment of a murine allotransplantation system of neonatal small intestine that recapitulates normal development.

To observe the developmental process of intestines under the environment without exposure to nutrient and digestive activity, the neonatal small intestine was harvested from *Lgr5*$^{EGFP\text{-}IRES\text{-}CreERT2/+}$ mice and transplanted into the kidney subrenal capsule of 8-week-old C57BL/6 recipients. The transplanted small intestine developed in a comparable manner with naïve counterparts. P; postnatal day.

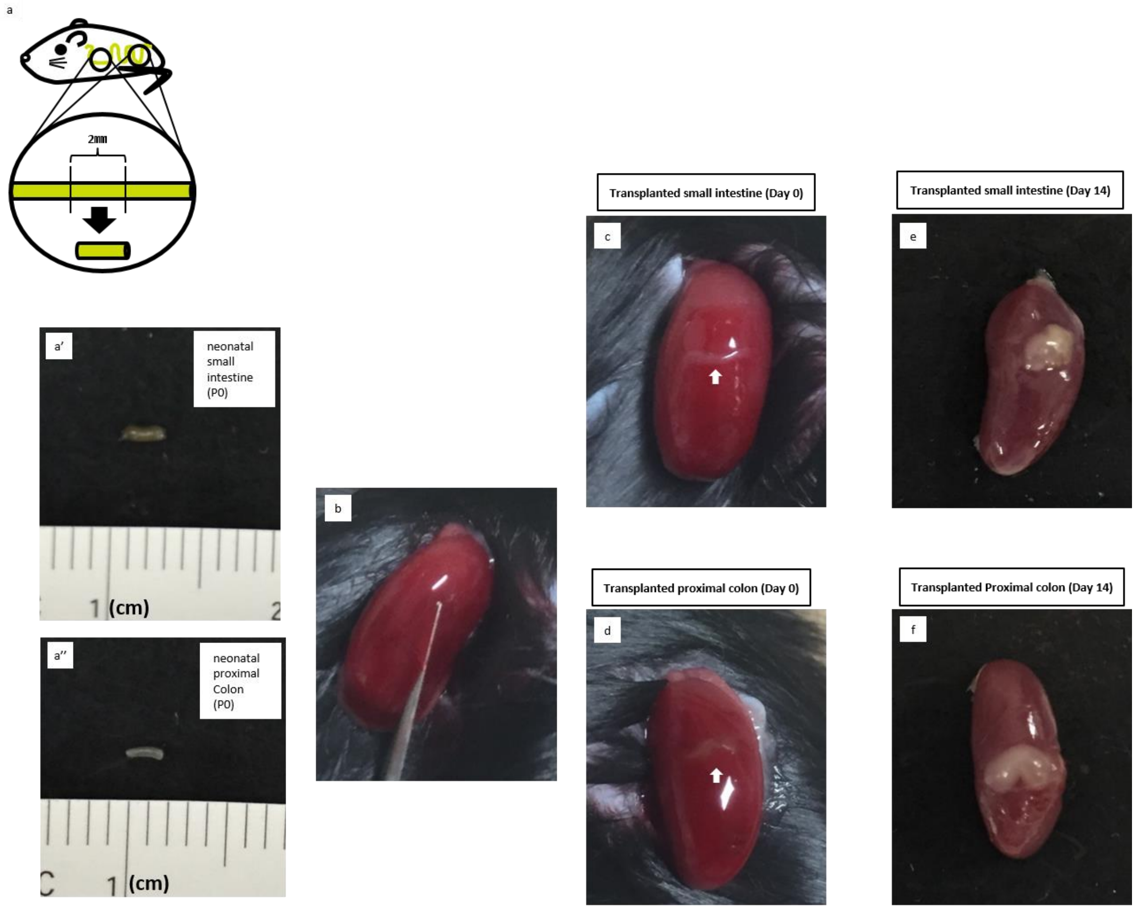

Supplementary Figure 6. Procedure for transplantation of the neonatal small intestine or proximal colon into kidney subrenal capsule.

(a-a") Small intestine (a') and proximal colon (a") tissues were dissected from P0 *Lgr5*$^{EGFP\text{-}Ires\text{-}CreERT2/+}$ mice (a). The length of the cut tissues was 2mm (longitudinal).

(b) A subcapsular pocket was created.

(c, d) Representative images of transplanted intestines after surgery. Small intestine (c) (c: white arrow) and proximal colon (d) (d: white arrow).

(e, f) Representative images of transplanted intestines 14 days after transplantation. Small intestine (e) and proximal colon (f).

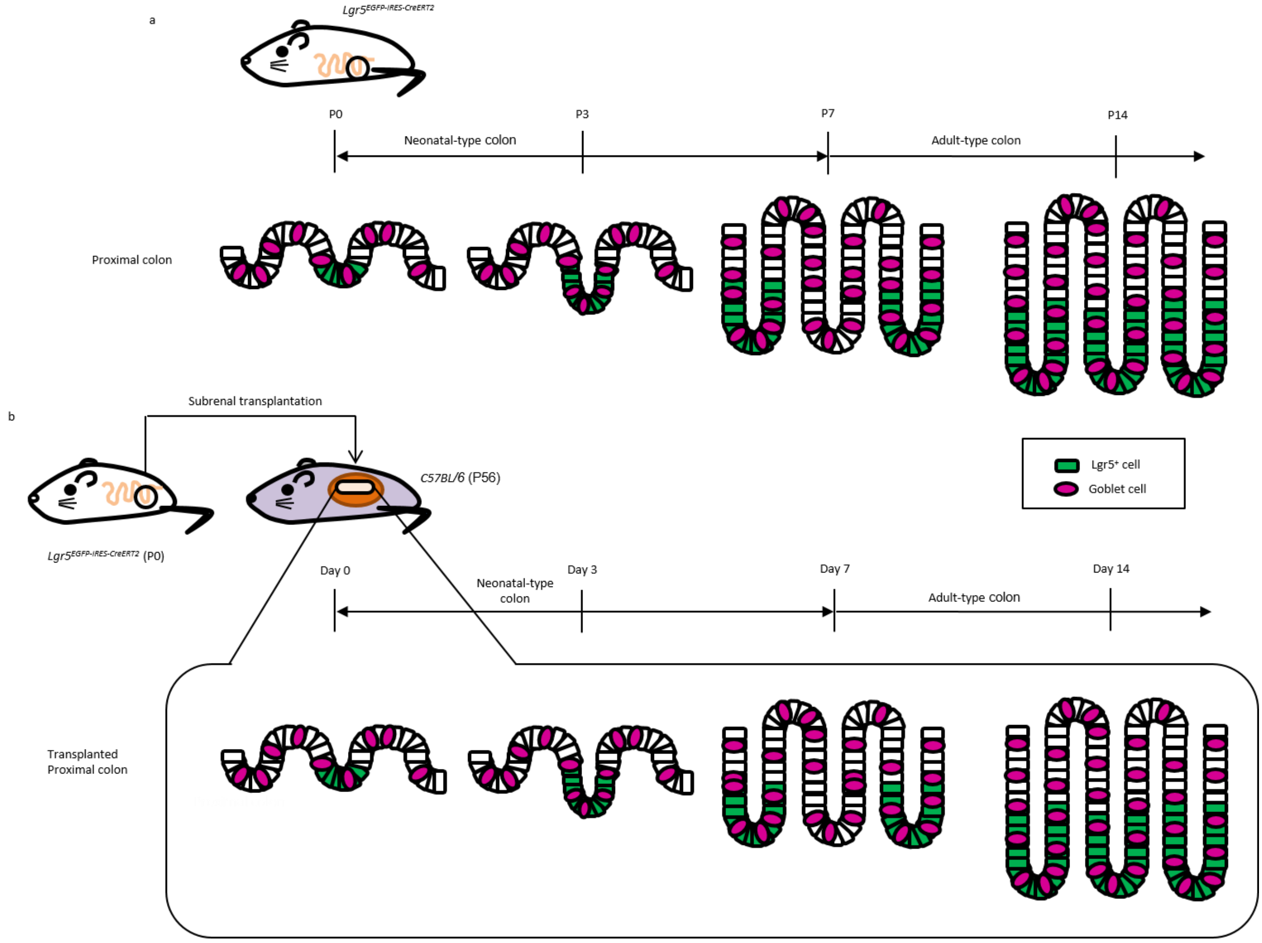

Supplementary Figure 7. Schematic representation: Murine allotransplantation system of the neonatal colon is established, which recapitulates normal development.

a. Schematic representation of the developmental process of the normal colon. When *Lgr5*$^{EGFP\text{-}IRES\text{-}CreERT2/+}$ mice are analyzed, the expression of Lgr5 can be detected as immunostaining for GFP. P; postnatal day.

b. To observe the developmental process of intestines under the environment without exposure to nutrient and digestion activity, the neonatal colon harvested from *Lgr5*$^{EGFP\text{-}IRES\text{-}CreERT2/+}$ mice were transplanted into kidney subrenal capsule of 8-week-old host *C57BL/6* mice. The transplanted colon developed in a comparable manner with naive mice. P; postnatal day.

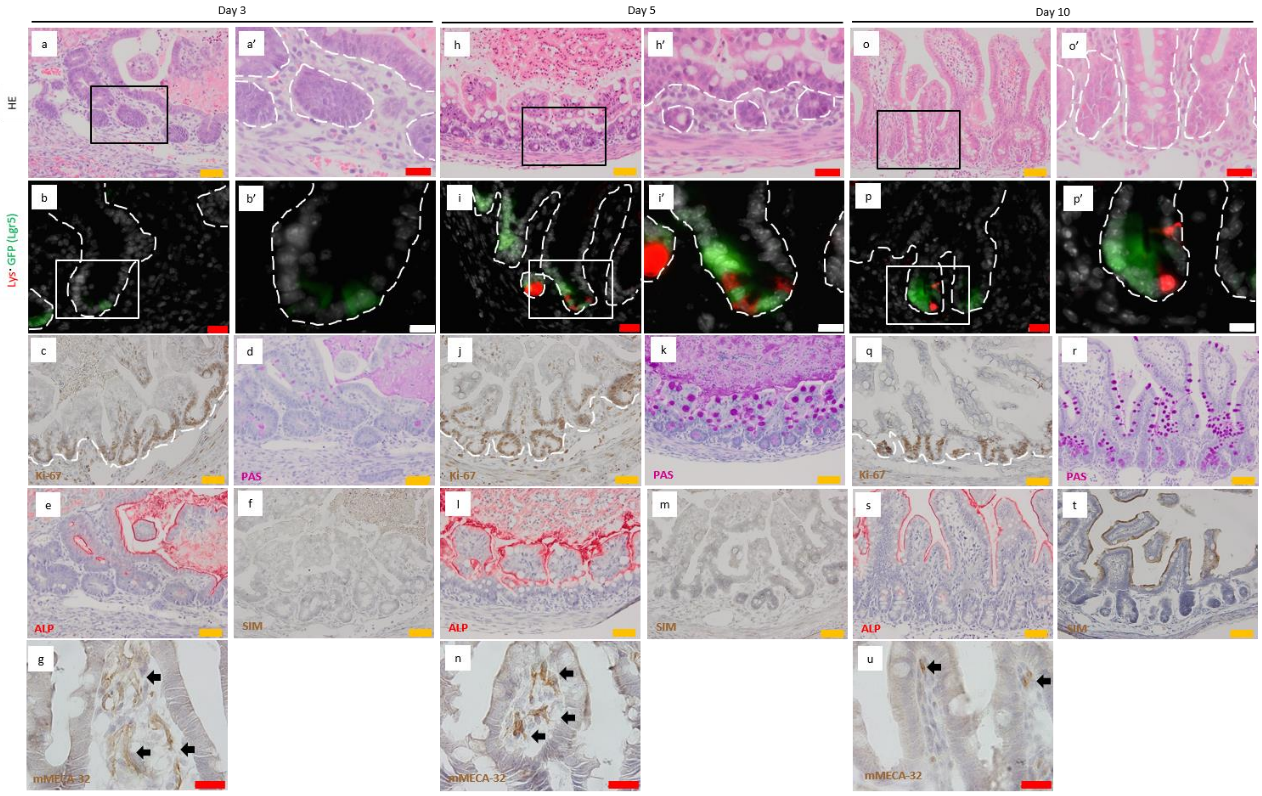

Supplementary Figure 8. Subrenal capsule can support the development of the transplanted neonatal small intestine to complete maturation.

(a, a’, h, h’, o, o’) H&E staining for transplanted small intestine harvested from P0 *Lgr5*$^{EGFP\text{-}IRES\text{-}CreERT2/+}$ mice at the the indicated days after transplantation.

(b, b’, i, i’, p, p’) Lysozyme staining (red) on sections of transplanted small intestine harvested from P0 *Lgr5*$^{EGFP\text{-}IRES\text{-}CreERT2/+}$ mice at the indicated days after transplantation, where the expression of Lgr5 was detected as GFP fluorescence (green).

(c, j, q) Immunostaining for Ki-67 (brown) as a marker of intestinal cell proliferation, on sections of transplanted small intestine derived from P0 *Lgr5*$^{EGFP\text{-}IRES\text{-}CreERT2/+}$ mice at the indicated days after transplantation.

(d, k, r) Periodic acid-Schiff (PAS) staining for transplanted small intestine harvested from P0 *Lgr5*$^{EGFP\text{-}IRES\text{-}CreERT2/+}$ mice at the indicated days after transplantation.

(e, l, s) Alkaline phosphatase staining for transplanted small intestine harvested from P0 *Lgr5*$^{EGFP\text{-}IRES\text{-}CreERT2/+}$ mice at the indicated days after transplantation.

(f, m, t) Immunostaining of transplanted intestinal tissues for brush border enzyme; sucrose-isomaltase (SIM) demonstrated functional maturation of them.

(g, n, u) Immunostaining for mMECA-32 (brown) (black arrows), as a marker of vasculature ingrowth, on sections of transplanted small intestine derived from P0 *Lgr5*$^{EGFP\text{-}IRES\text{-}CreERT2/+}$ mice at the indicated days after transplantation.

Higher magnification images (a’, b’, h’, i’, o’, p’) of the corresponding boxed area are shown below the respective images. White dashed lines indicate the boundary between epithelium and lamina propria. Scale bars: orange, 50 μm; red, 20 μm; white, 10 μm.

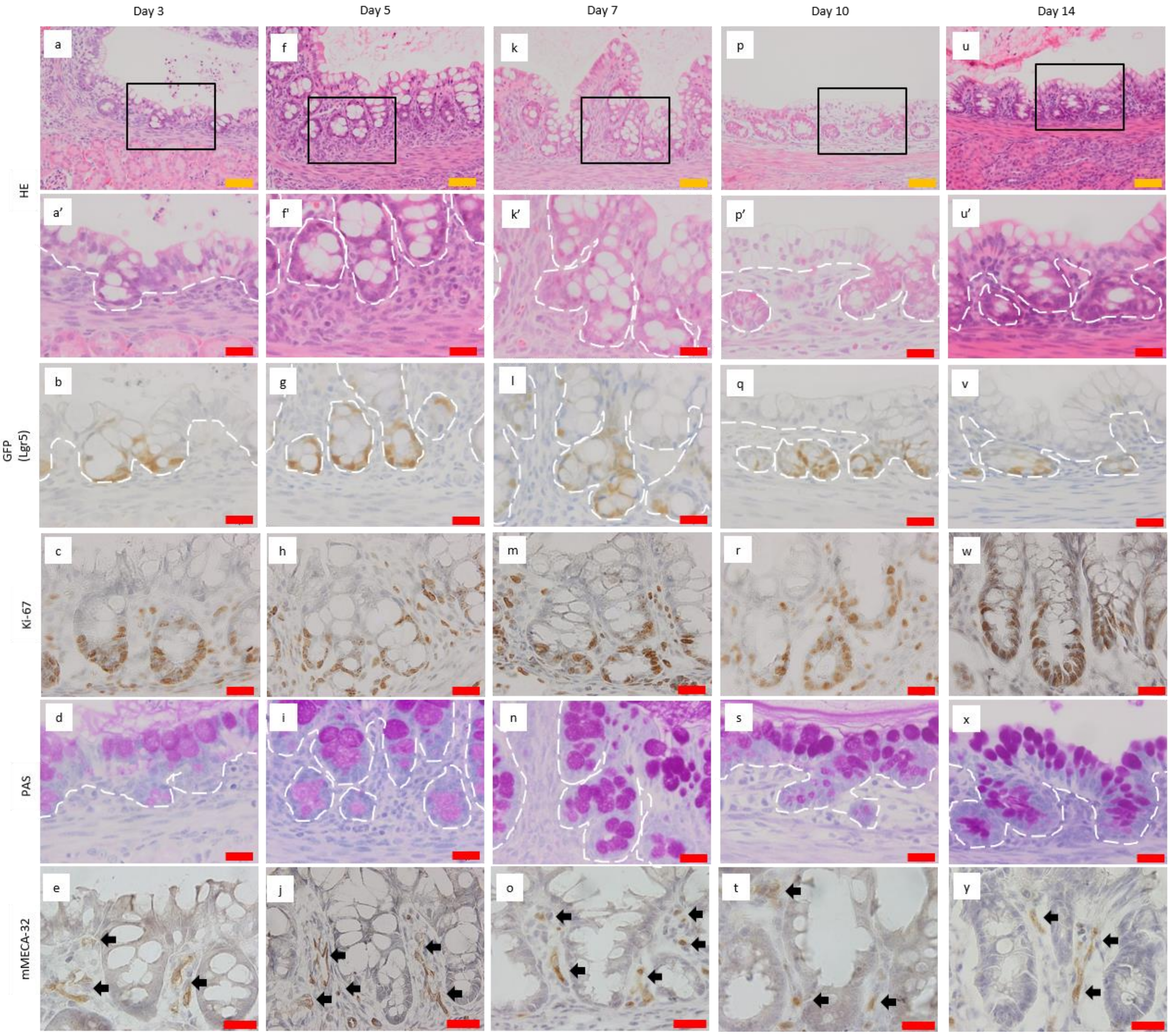

Supplementary Figure 9. Subrenal capsule can support the development of transplanted neonatal colon to complete maturation.

(a, a’, f, f’, k, k’, p, p’, u, u’) H&E staining for transplanted colon derived from P0 *Lgr5*$^{EGFP\text{-}IRES\text{-}CreERT2/+}$ mice at the indicated days after transplantation.

(b, g, l, q, v) Immunostaining of GFP (brown) to detect the expression of Lgr5 using colon of *Lgr5*$^{EGFP\text{-}IRES\text{-}CreERT2/+}$ mice at the indicated days after transplantation.

(c, h, m, r, w) Immunostaining for Ki-67 (brown), as a marker of intestinal cell proliferation, on sections of transplanted colon derived from P0 *Lgr5*$^{EGFP\text{-}IRES\text{-}CreERT2/+}$ at the indicated days after transplantation.

(d, i, n, s, x) Periodic acid-Schiff (PAS) staining for transplanted colon derived from P0 *Lgr5*$^{EGFP\text{-}IRES\text{-}CreERT2/+}$ mice at the indicated days after transplantation.

(e, j, o, t, y) Immunostaining for mMECA-32 (brown) (black arrows), as a marker of vasculature ingrowth, on sections of transplanted colon derived from P0 *Lgr5*$^{EGFP\text{-}IRES\text{-}CreERT2/+}$ mice at the indicated days after transplantation.

Higher magnification images (a’, f’, k’, p’, u’) of the corresponding boxed area are shown below the respective images. White dashed lines indicate the boundary between epithelium and lamina propria. Scale bars: orange, 50 µm; red, 20 µm.

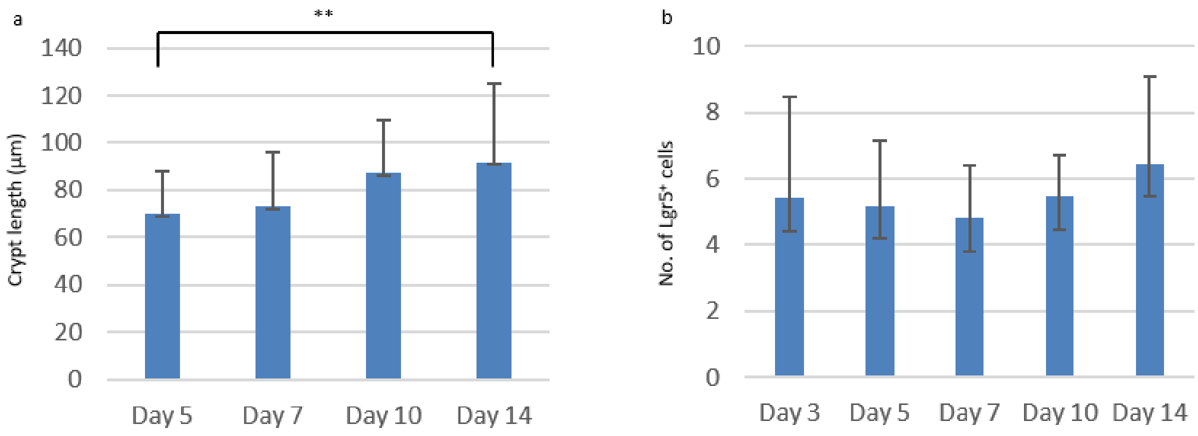

Supplementary Figure 10. The crypt length and the number of Lgr5-EGFP^{+} cells of transplanted colon

(a) The crypt length of transplanted colon harvested from P0 *Lgr5*$^{EGFP\text{-}IRES\text{-}CreERT2/+}$ mice at the indicated days after transplantation. The number of crypts analyzed were as follows: Day 5; n = 47 in 4 transplants; Day 7; n = 45 in 4 transplants, Day 10; n = 41 in 3 transplants, and Day 14; n = 56 in 5 tranplants. These transplanted intestines were derived from 4 mice (Day 5), 2 mice (Day 7), 2 mice (Day 10), and 3 mice (Day 14), respectively.

(b) The number of Lgr5-EGFP^{+} cells per crypt. The numbers of crypts analyzed were as follows: Day 3; n = 32 in 2 transplants, Day 5; n = 40 in 4 transplants; Day 7; n = 41 in 4 transplants, Day 10; n = 35 in 3 transplants, and Day 14; n = 39 in 5 transplants. These transplanted intestines were derived from 2 mice (Day 3), 4 mice (Day 5), 2 mice (Day 7), 2 mice (Day 10), and 3 mice (Day 14), respectively. Error bars indicate standard deviation.

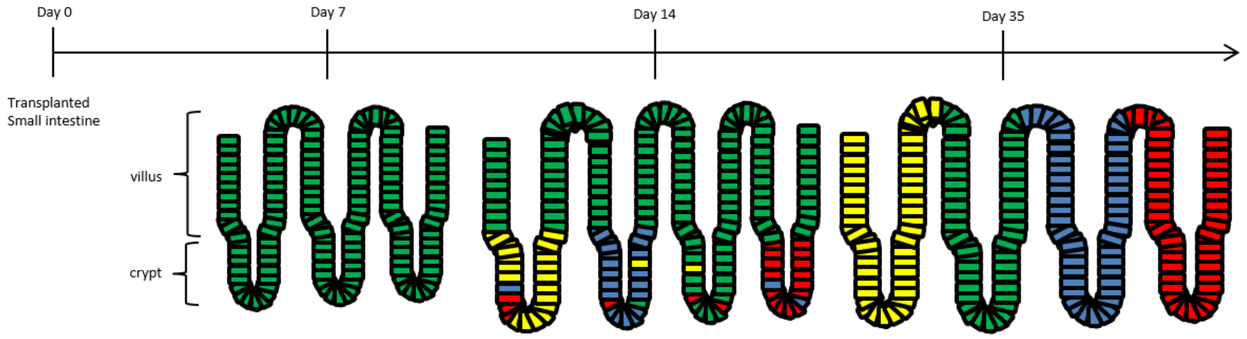

Supplementary Figure 11. Graphical explanation of the results when P0 small intestine of *Lgr5*$^{EGFP\text{-}IRES\text{-}CreERT2/+}$; *Rosa26*$^{rbw/+}$ mice were transplanted into subrenal capsule followed by tamoxifen induction

By means of intraperitoneal injection of tamoxifen into the host mice, CreERT2-mediated recombination, followed by random expression of mCerulean, mCherry, or mOrange was induced in Lgr5-expressing stem cells of the transplanted neonatal intestine. At 7 days after tamoxifen induction (Day 14), it was revealed that the crypts contained colored cells, demonstrating the presence of descendants of Lgr5^{+} stem cells. At 28 days after tamoxifen induction (Day 35), crypt-villus units in the small intestine were shown to be composed of cells of a single color.

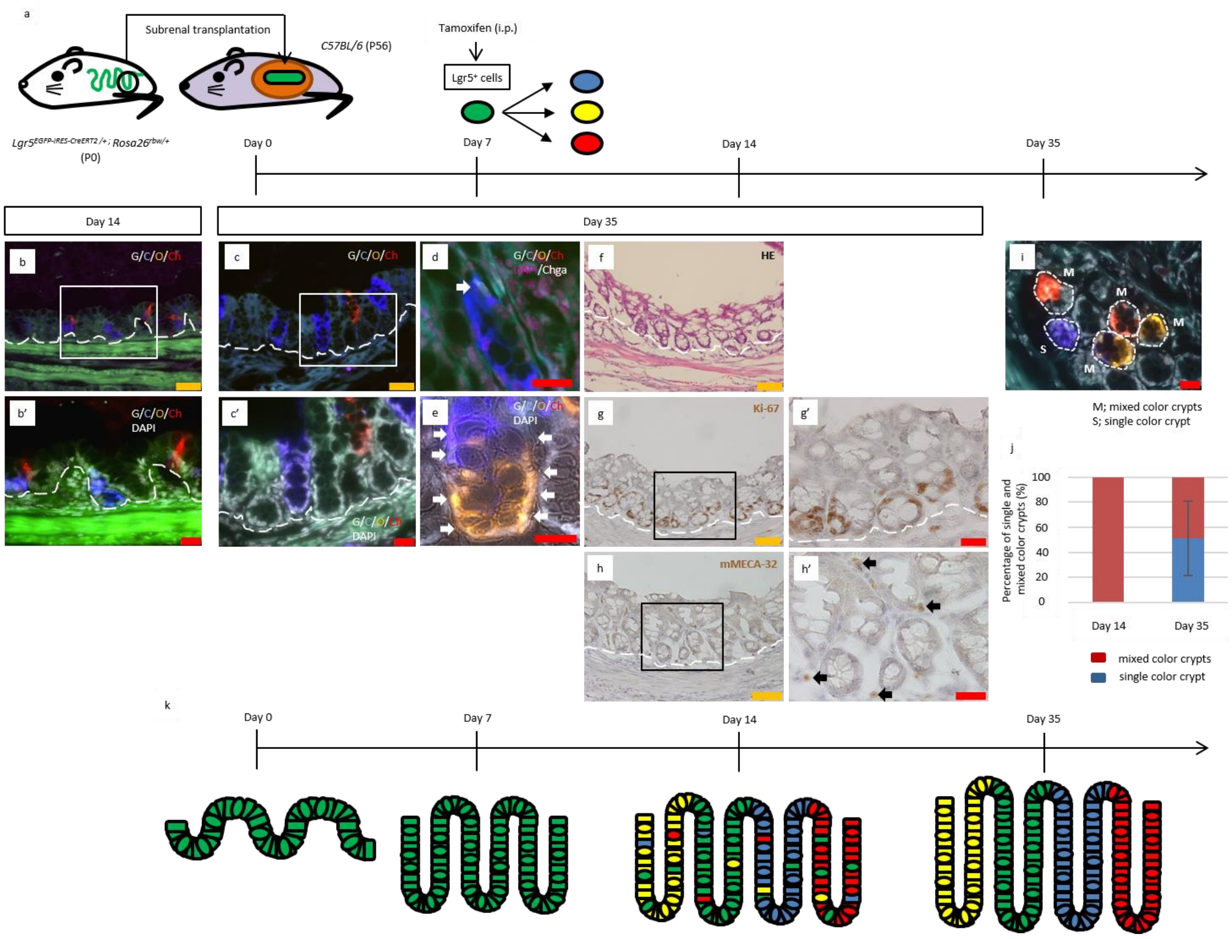

Supplementary Figure 12. Multicolor lineage tracing of Lgr5⁺ cells revealed that the intestinal stem cells at P7 can autonomously differentiate into all types of cells that configure the whole crypt of colon.

(a) Schematic protocol of transplanting neonatal colon into subrenal capsule followed by tamoxifen induction at 7 days after transplantation and harvest at 14 or 35 days after transplantation. P; postnatal day.

(b, c). Fluorescent images of transplanted colon, where multicolor labelling was induced in Lgr5⁺ cells, followed by lineage tracing until day 14 (b) or day 35 (c). The number of transplants analyzed were as follows: day 14; n = 4, and day 35; n = 8. These transplanted intestines were derived from 2 mice (Day 14) and 4 mice (Day 35), respectively.

(d) Co-staining of the single-colored cluster for markers of differentiated lineages demonstrated that transplanted colon differentiatied into enteroendocrine cells (d) (d: white arrow).

(e) Representative image of Lgr5⁺ cell-derived clone that contained goblet cells, in which apical localization of secretory granules and compressed nuclei toward basal direction were observed (white arrows).

(f) H&E staining for transplanted colon harvested from P0 *Lgr5*$^{CreERT2/+}$ *Rosa26*$^{rbw/+}$ mice at day 35 after transplantation.

(g, g') Immunostaining for Ki-67 (brown), as a marker of intestinal cell proliferation, on sections of transplanted colon derived from P0 *Lgr5*$^{EGFP\text{-}IRES\text{-}CreERT2/+}$ *; Rosa26*$^{rbw/+}$ mice at the indicated days after transplantation.

(h, h') Immunostaining for mMECA-32 (brown) (i': black arrows), as a marker of vasculature ingrowth, on sections of transplanted colon derived from P0 *Lgr5*$^{EGFP\text{-}IRES\text{-}CreERT2/+}$ *; Rosa26*$^{rbw/+}$ mice at the indicated days after transplantation.

(b', c', g', h') High-power field images of b, c, h, and i, respectively.

(i) Representative images of the horizontal section of transplanted colon. Day 35 grafts were analyzed. M; mixed color crypts, S; single color crypts.

(j) The percentage of single and mixed color crypts. The numbers of crypts analyzed were as follows: day 14; n = 39 in 4 transplants and day 35; n = 65 in 8 transplants. The transplanted intestines were derived from 2 mice (Day 14) and 4 mice (Day 35). Error bars indicate standard deviation.

(k) Graphical explanation of developmental process observed in neonatal-type and adult-type colon of *Lgr5* $^{EGFP\text{-}IRES\text{-}CreERT2/+}$*; Rosa26*$^{rbw/+}$ mice.

White dashed lines indicate the boundary between epithelium and lamina propria. Scale bars: orange, 50 μm; red, 20 μm.

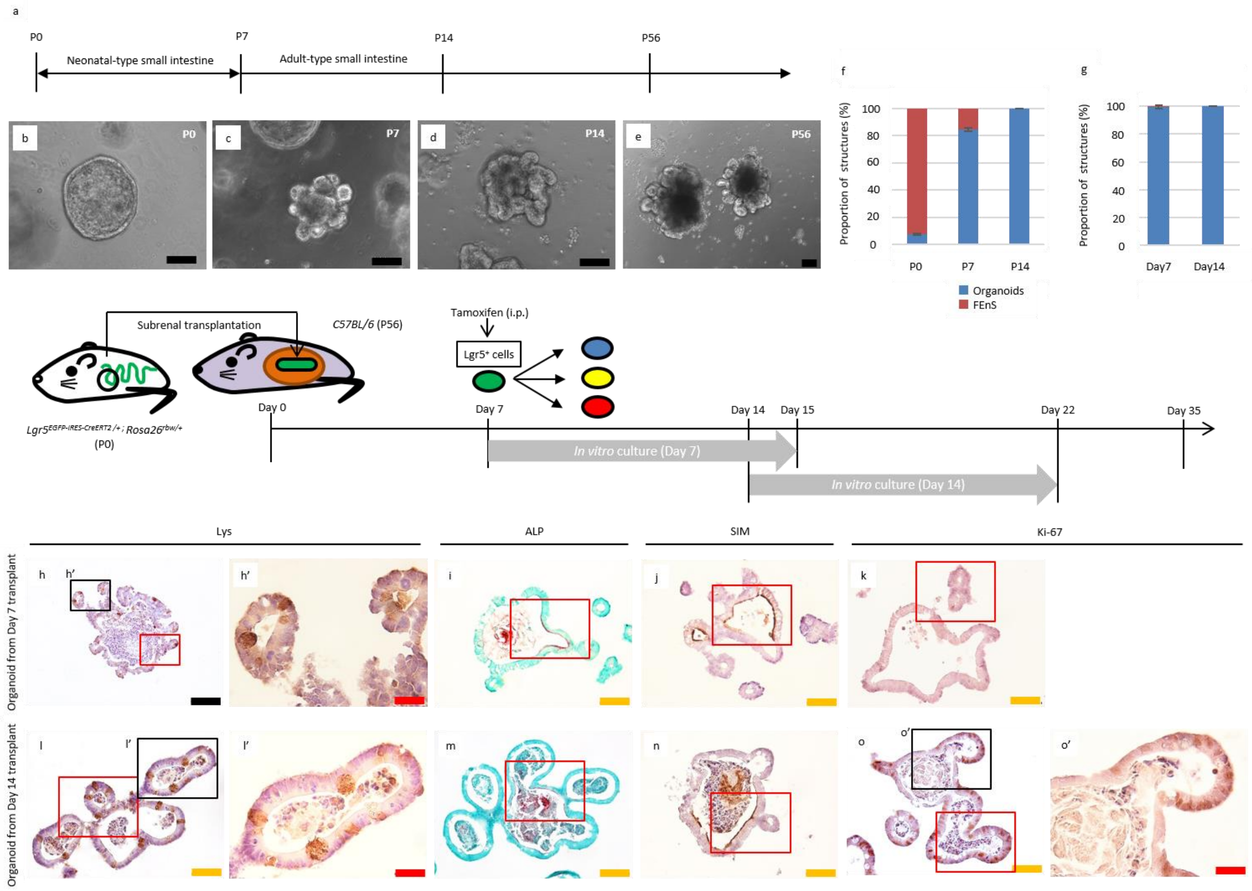

Supplementary Figure 13. *In vitro* culture of transplanted small intestines demonstrated that they contained the crypts that could form organoids rather than FEnS, which are derived from immature epithelial cells.

(a) Developmental process of small intestine; our results indicated that intestine from P0 to P7 exhibited neonatal-type feature, whereas that after P7 showed features comparable with those of an adult.

(b) Representative image of the Fetal Enterospheres (FEnS), which had been expanded after an 8-day long culture, from epithelial cells harvested from the proximal regions of the small intestine in P0 *C57BL/6* mice.

(c, d, e) Representative images of the organoids, which had been expanded after an 8-day long culture, from the crypts harvested from proximal regions of the small intestine in P7 (c), P14 (d), and P56 (e) *C57BL/6* mice. The features of the organoids rather than spherical shape demonstrated that these organoids were derived from matured crypts.

(f) The emergence rate of FEnS and organoids from the proximal regions of the small intestine at the indicated birth dates. P0; n = 3, P7; n = 2, P14; n = 3.

(g) The emergence rate of FEnS and organoids from the transplanted proximal regions of the P0 small intestine at the indicated days after transplantation. Day 7; n = 5, Day 14; n = 5.

(h-o’) Grafts were harvested at 7 days (h-k) or 14 days (l-o’) after transplantation and their crypts were served for *in vitro* culture. Formed organoids at day 8 were analyzed.

(h, h’, l, l’) Representative images of immunostaining of organoids for Lys. Brown-stained cells indicate Lys-positive Paneth cells.
(i, m) Representative images of alkaline phosphatase staining of organoids. Red-stained blush border enzyme indicated that cultured organoids had expanded, differentiating into matured villi. Nuclei were stained using methyl-green.
(j, n) Immunostaining of organoid revealing maturation in the cells that expressed brush border enzyme, sucrose-isomaltase (SIM).
(k, o, o’) Immunostaining for Ki-67 (brown) as a marker for cell proliferation.
(h’, l’, o’) Higher magnification images of the boxed area (black) in the corresponding images (h, l, o) are shown below the respective images. Higher magnification images of the boxed area (red) in h-o are also shown in Figure. 4o-r, 4t-w, respectively.
Scale bars: black, 100 μm; orange, 50 μm; red, 20 μm.
